# Outer membrane permeabilization by the membrane attack complex sensitizes Gram-negative bacteria to antimicrobial proteins in serum and phagocytes

**DOI:** 10.1371/journal.ppat.1009227

**Published:** 2021-01-22

**Authors:** Dani A. C. Heesterbeek, Remy M. Muts, Vincent P. van Hensbergen, Pieter de Saint Aulaire, Tom Wennekes, Bart W. Bardoel, Nina M. van Sorge, Suzan H. M. Rooijakkers

**Affiliations:** 1 Department of Medical Microbiology, University Medical Center Utrecht, Utrecht University, Utrecht, The Netherlands; 2 Department of Chemical Biology and Drug Discovery, Utrecht Institute for Pharmaceutical Sciences and Bijvoet Center for Biomolecular Research, Utrecht University, Utrecht, The Netherlands; University of Toronto, CANADA

## Abstract

Infections with Gram-negative bacteria form an increasing risk for human health due to antibiotic resistance. Our immune system contains various antimicrobial proteins that can degrade the bacterial cell envelope. However, many of these proteins do not function on Gram-negative bacteria, because the impermeable outer membrane of these bacteria prevents such components from reaching their targets. Here we show that complement-dependent formation of Membrane Attack Complex (MAC) pores permeabilizes this barrier, allowing antimicrobial proteins to cross the outer membrane and exert their antimicrobial function. Specifically, we demonstrate that MAC-dependent outer membrane damage enables human lysozyme to degrade the cell wall of *E*. *coli*. Using flow cytometry and confocal microscopy, we show that the combination of MAC pores and lysozyme triggers effective *E*. *coli* cell wall degradation in human serum, thereby altering the bacterial cell morphology from rod-shaped to spherical. Completely assembled MAC pores are required to sensitize *E*. *coli* to the antimicrobial actions of lysozyme and other immune factors, such as Human Group IIA-secreted Phospholipase A2. Next to these effects in a serum environment, we observed that the MAC also sensitizes *E*. *coli* to more efficient degradation and killing inside human neutrophils. Altogether, this study serves as a proof of principle on how different players of the human immune system can work together to degrade the complex cell envelope of Gram-negative bacteria. This knowledge may facilitate the development of new antimicrobials that could stimulate or work synergistically with the immune system.

## Author summary

In this paper we identified how different players of the human immune system cooperate to degrade the complex cell envelope of Gram-negative bacteria. The outer membrane of Gram-negative bacteria forms an impermeable barrier for various antimicrobial proteins of the immune system. Here we show that complement-dependent Membrane Attack Complex (MAC) formation permeabilizes this barrier, allowing otherwise impermeable antimicrobial proteins to reach their targets underneath the outer membrane. Specifically, we show that outer membrane damage by the MAC allows lysozyme to degrade the peptidoglycan layer, and secreted phospholipase A_2_-IIA to hydrolyze the bacterial inner membrane. MAC formation also sensitizes Gram-negative bacteria to more efficient degradation and killing inside human neutrophils. Altogether, this knowledge may guide the development of new antimicrobial strategies to treat infections caused by Gram-negative bacteria.

## Introduction

Infections with Gram-negative bacteria form a major problem for human health, which is mainly due to the increase in antibiotic resistance. According to the World Health Organization there is an urgent need for alternative strategies to treat infections with Gram-negative bacteria such as *Acinetobacter baumanni*, *Pseudomonas aeruginosa* and *Escherichia coli (E*. *coli)*, which are at the top of the priority list of antibiotic*-*resistant bacterial species [1]. The cell envelope of Gram-negative bacteria consists of an inner membrane, a peptidoglycan layer and an additional outer membrane [2]. The peptidoglycan layer of bacteria is important for the maintenance of osmotic balance and to maintain the bacterial cell shape, for example rod-shaped for *E*. *coli* [3]. The outer membrane forms a physical barrier to a large number of antimicrobial compounds [4], which makes it challenging to develop new antibiotics against these bacteria. Combination therapy of antibiotics and outer membrane permeabilizing agents have become more attractive over the last decades [5–9]. Furthermore, there is increased awareness that antibiotics may be more effective in the presence of the human immune system [10], which has evolved strategies to disrupt the complex cell envelope of Gram-negative bacteria. Increased understanding of these mechanisms may facilitate the development of new antimicrobials, that could stimulate or work synergistically with the immune system.

The human body fights invading bacteria via cellular and humoral immune components. Cellular protection is provided by immune cells, such as neutrophils, that engulf bacteria and expose them to a large number of antimicrobial compounds [11,12]. One of these intracellular proteins is lysozyme, a 14.7 kDa protein that degrades bacterial peptidoglycan [12,13]. Additionally, lysozyme is present in bodily fluids such as the blood, saliva and tears [13]. Although lysozyme and other (intracellular) antimicrobials can efficiently act on Gram-positive bacteria [14], many of these are considered to be inactive against Gram-negative bacteria [15,16], partly because they cannot cross the bacterial outer membrane. Humoral innate immunity against bacteria is mainly dependent on the complement system, which consists of a protein network circulating in the blood. Complement activation triggers the deposition of C5 convertases on the bacterial surface that cleave C5 into C5a and C5b. C5b associates with C6, C7, C8 and multiple copies of C9 to form large ring-structured pores (C5b-9) [17,18] called Membrane Attack Complexes (MACs). It has long remained unclear how MAC pores damage the complex cell envelope of Gram-negative bacteria in such a way that this leads to bacterial cell death. Furthermore, although the bactericidal activity of the MAC has been analyzed in combination with other immune factors [19,20], tools to study these processes at a molecular level were limiting.

We recently developed a fluorescent reporter system to distinguish between outer and inner membrane perforation in Gram-negative bacteria by flow cytometry. Specifically, *E*. *coli* was genetically engineered to express mCherry in the periplasm and GFP in the cytoplasm. Leakage of these proteins and influx of impermeable DNA dyes functioned as a detailed readout for membrane damage. Using these tools, we demonstrated that the MAC efficiently permeabilizes the bacterial outer membrane which can, after a delay, also trigger destabilization of the bacterial inner membrane [10,18]. Whereas outer membrane damage by itself is not sufficient to kill Gram-negative bacteria, inner membrane damage is crucial to prevent colony formation. By studying how MAC pores affect membrane integrity using purified complement proteins, we also noticed that the MAC can efficiently kill Gram-negative bacteria, but does not trigger leakage of cytoplasmic proteins (GFP). Furthermore, MAC pores do not alter the cell shape of bacteria [10,18], suggesting that the peptidoglycan layer remains intact. This suggests that although the MAC is able to directly kill Gram-negative bacteria, other factors are required to further degrade the bacterial cell envelope.

Here we demonstrate, at a molecular level, how MAC-dependent outer membrane damage enhances the susceptibility of *E*. *coli* to antimicrobial proteins with different effector functions, such as human lysozyme and Group IIA secreted phospholipase A2 (hGIIA)[21]. Lysozyme turned out to be the crucial factor for the disintegration of the cell wall of *E*. *coli* in serum. In addition, we show that MAC-dependent outer membrane damage enhances killing and degradation of bacteria inside human neutrophils, suggesting that it sensitizes bacteria to antimicrobial proteins in the phagolysosome. Altogether, this study serves as a proof of principle on how different components of the immune system can act synergistically to effectively clear invading bacteria.

## Results

### The MAC and lysozyme trigger *E*. *coli* cell wall degradation in human serum

**Fig 1:**
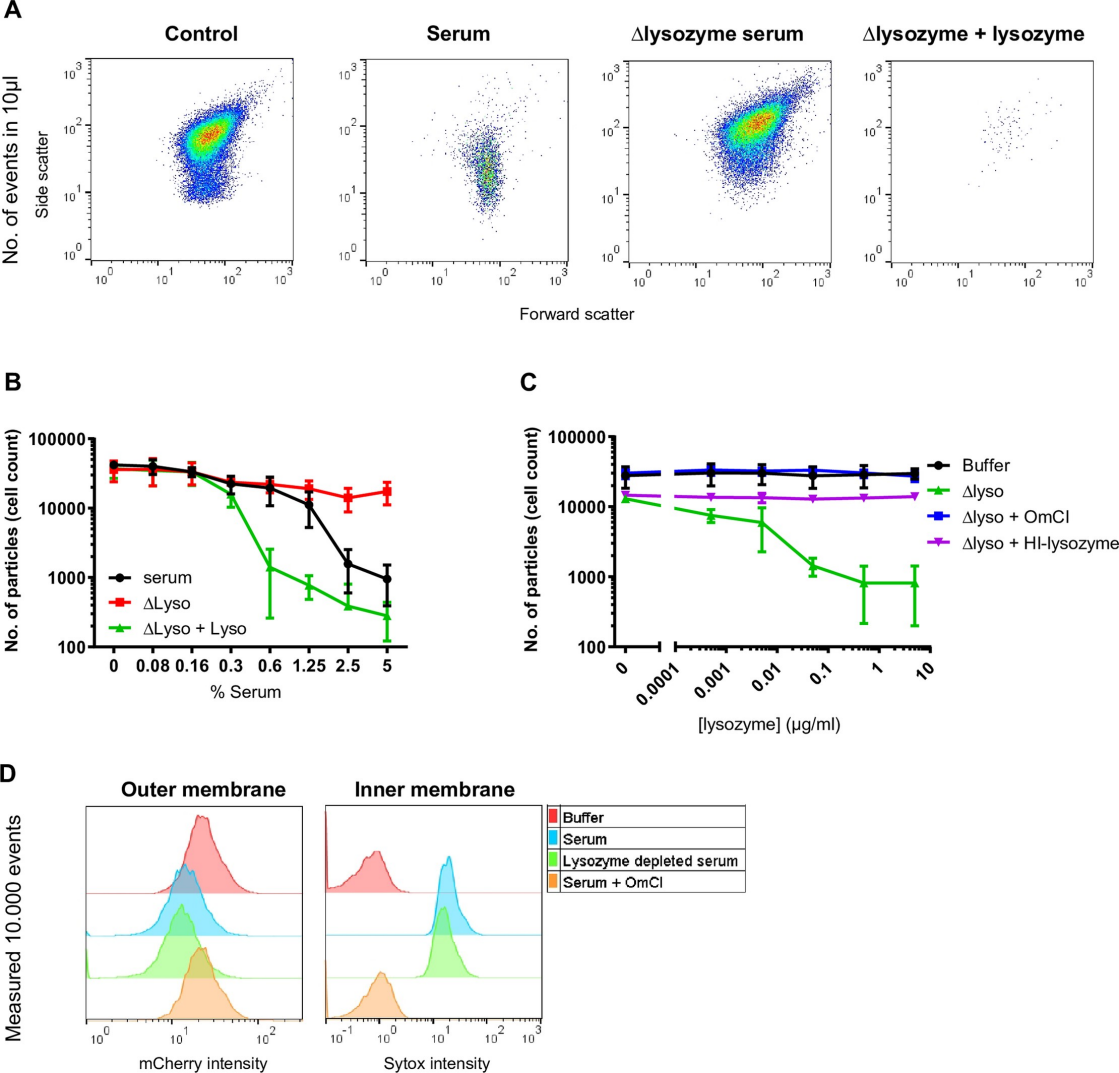
The MAC and lysozyme trigger *E*. *coli* cell wall degradation in human serum. **A**) Flow cytometry plots (FSC/SSC) of the number of *E*. *coli* particles in 10 μl after exposure to buffer, 5% serum or Δlysozyme serum with or without 5 μg/ml lysozyme for 60 minutes at 37°C. A SSC threshold was set based on untreated bacteria to filter out background noise and to determine changes in FSC/SSC upon treatment with different serum conditions. **B**) Cell count in 10 μl incubation volume of *E*. *coli* treated with a concentration range of nhs or Δlysozyme serum with or without 5 μg/ml lysozyme for 60 min 37°C. The number of cells represent the events in 10 μl sample that were measured within the conditions depicted in **A**. **C**) Cell count in 10 μl incubation volume of *E*. *coli* treated with buffer or 5% Δlysozyme serum in the presence or absence of 20 μg/ml OmCI and a titration of lysozyme or heat inactivated (HI) lysozyme for 60 min 37°C. **D**) Flow cytometry histograms of outer membrane damage (left: mCherry) and inner membrane damage (right: Sytox blue) of bacteria treated with buffer, 1% serum with or without 20 μg/ml OmCI or 1% Δlysozyme serum. **A, D**) Flow cytometry plots and histograms represent data of three independent experiments. **B, C**) Data represent mean ±SD of 3 independent experiments.

In previous studies, we investigated how the MAC affects the cell wall integrity and viability of *E*. *coli* using purified complement components. We observed that the MAC efficiently damages the outer and inner membrane of bacteria, which triggers bacterial cell death. Nevertheless, confocal microscopy images revealed that MAC-treated bacteria remain rod-shaped, which is in line with the fact that these bacteria retained their forward and side scatter when measured by flow cytometry [10,18]. In contrast to these purified conditions, we noticed that *E*. *coli* bacteria started to disappear from the flow cytometry gate after approximately 40–60 minutes when they were incubated with 5% human serum (**Fig 1A**). To investigate this further, we adjusted the acquisition settings of our flow cytometry experiment to quantify the number of particles in a sample, by measuring a fixed volume. Particles were qualified as rod-shaped bacteria when their forward scatter (FSC) and side scatter (SSC) was similar to that of untreated bacteria (**Fig 1A; control**). A threshold was set on the SSC, to prevent the detection of background events caused by serum. Exposing bacteria to serum drastically decreased the number of detected particles, suggesting that these bacteria lost their natural rod-shape (**Fig 1A** and **1B**). Since the bacterial peptidoglycan layer is important in maintaining the bacterial cell shape and to prevent bacterial lysis from turgor pressure [22], we questioned whether lysozyme in serum could be responsible for the observed loss of particles. Although lysozyme is normally not active against *E*. *coli*, we hypothesized that the combined action of complement and lysozyme in serum might promote the degradation of the bacterial cell wall. To test this, we depleted >99% of the endogenous lysozyme from our serum pool (Δlysozyme serum). Whereas the lysozyme concentration in serum is ~1–2 μg/ml, this is only ~0.01 μg/ml in Δlysozyme serum (**S1A and S1B Fig and S1 Table**). When bacteria were treated with Δlysozyme serum, there was no major shift in their FSC/SSC compared to untreated bacteria, suggesting that these bacteria retained their natural shape (**Fig 1A** and **1B**). When 5 μg/ml purified lysozyme was added to the Δlysozyme serum, we observed even more efficient particle loss than in non-depleted serum (**Fig 1A** and **1B**), likely because this lysozyme concentration exceeds that of 5% normal serum by a factor 100. We next titrated lysozyme into 5% Δlysozyme serum to validate whether physiological concentrations of lysozyme can also restore the effect of Δlysozyme serum (**Fig 1C**). Here, we observed a dose-dependent increase in particles loss, in which lysozyme was already functional at concentrations of 0.05 μg/ml, which is comparable to the concentration that is naturally present in 5% Δlysozyme serum (**Fig 1C**). The efficacy of lysozyme in serum was dependent on the presence of MAC pores, as no particles disappeared in the presence of the C5 inhibitor OmCI, which prevents MAC formation [23] (**Figs 1C** and **S1C**). Furthermore, the addition of heat-inactivated lysozyme to the Δlysozyme serum did not trigger any additional particle loss (**Fig 1C**). The slight decrease in particles that we observed in 5% Δlysozyme serum without adding extra lysozyme (**Fig 1C**) was dependent on the presence of MAC pores, since it was absent in the presence of the C5 inhibitor OmCI (**Fig 1C**). This is potentially caused by the leakage of periplasmic proteins or the influx of serum proteins and peptides through MAC pores.

Since we observed that lysozyme plays a critical role in changing the bacterial cell shape in serum, we next questioned whether lysozyme contributes to outer or inner membrane damage. Although we previously observed that lysozyme is not essential for inner membrane damage or killing of *E*. *coli* in 10% serum [18], it may play a more crucial role at lower serum concentrations. To study potential damage to each of the two bacterial membranes separately, we used the genetically engineered *E*. *coli* strain that expresses mCherry in the periplasm and GFP in the cytoplasm (_peri_mCherry/_cyto_GFP *E*. *coli* MG1655) [18]. In addition, we included a naturally impermeable DNA dye (Sytox) to measure inner membrane destabilization. We treated bacteria with 1% serum or Δlysozyme serum and measured the fluorescence intensity for the three markers. A reduced mCherry signal, an increase in Sytox intensity and a reduced viability was observed in the presence and in the absence of lysozyme, showing that lysozyme is not essential to damage both bacterial membranes of this *E*. *coli* strain in serum (**Figs 1D** and **S1D**). In contrast, outer and inner membrane damage caused by serum was dependent on the MAC, since both membranes remained intact and bacteria survived in the presence of OmCI (**Figs 1D** and **S1D**). Altogether, MAC-dependent damage to both bacterial membranes does not depend on lysozyme. However, lysozyme is essential to further degrade the bacterial cell envelope in serum, resulting in changes in FSC/SSC as measured by flow cytometry.

### The MAC and lysozyme in serum trigger alterations in the morphology of *E*. *coli*

**Fig 2:**
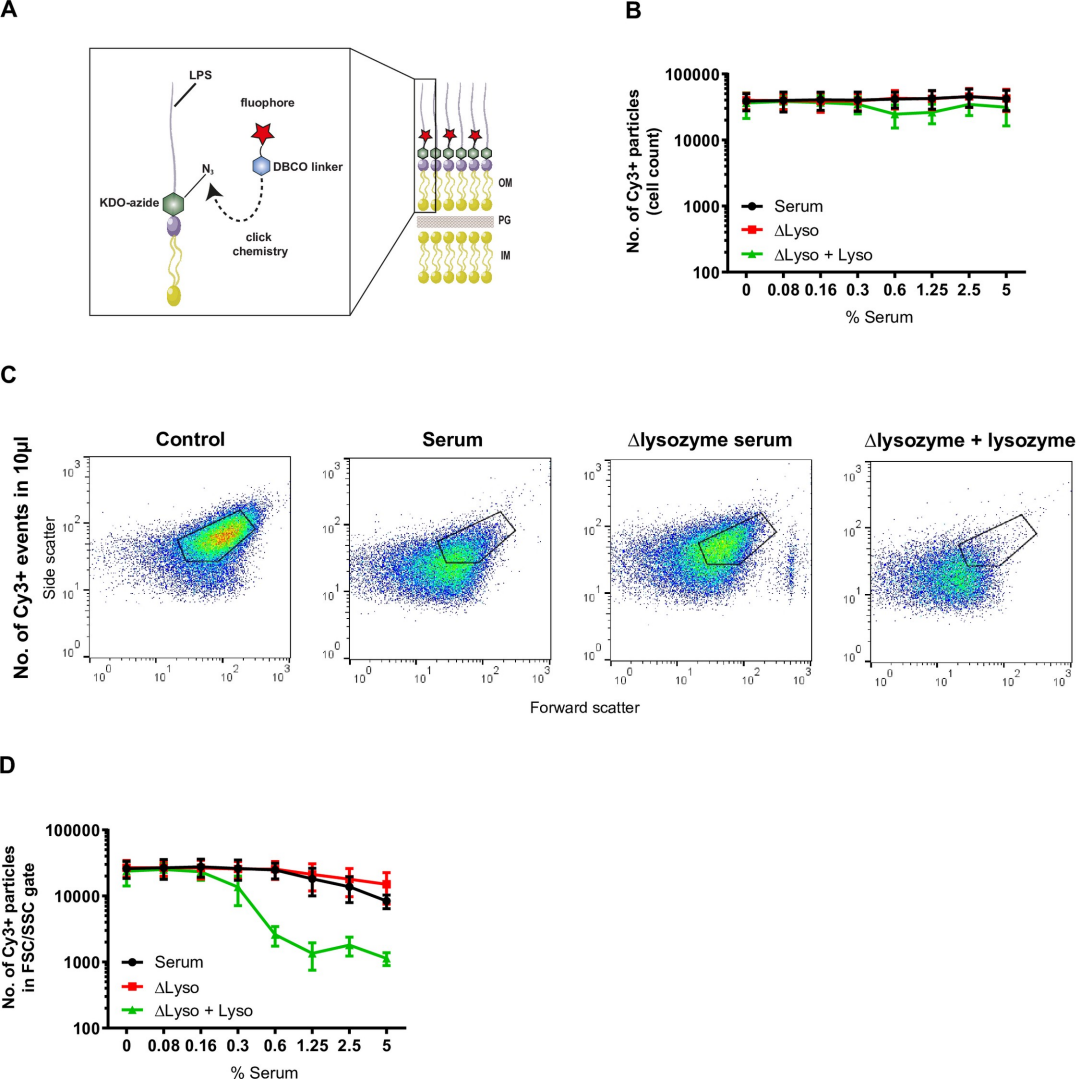
The MAC and lysozyme in serum trigger alterations in the morphology of *E*. *coli*. **A**) Schematic representation of Cy3-labeling of *E*. *coli* LPS via click chemistry. Bacteria are incubated with KDO-azide (green), which is incorporated into LPS. A DBCO (blue) linked to the fluorophore Cy3 (red) can subsequently react with the azide group via click chemistry. OM = outer membrane, PG = peptidoglycan, IM = inner membrane. **B**) The number of Cy3-positive *E*. *coli* cells in 10μl sample after exposure to a concentration range of serum or Δlysozyme serum with or without 5 μg/ml lysozyme. Samples were measured without a SSC threshold. **C**) Flow cytometry plots (FSC/SSC) of Cy3-positive *E*. *coli* particles in 10 μl sample after exposure to buffer, 5% nhs, or 5% Δlysozyme serum with or without 5 μg/ml lysozyme for 60 minutes at 37°C. **D**) Cy3-labeled *E*. *coli* was treated with a concentration range of serum or Δlysozyme serum with or without 5 μg/ml lysozyme for 60 min 37°C. For each condition, the number of Cy3-positive particles within the FSC/SSC gate of untreated bacteria was quantified in 10μl. **B-D**) The lysozyme concentration that was used exceeds the concentration in 5% serum by a factor 100. **B, D**) Data represent mean ±SD of 3 independent experiments. **C**) Flow cytometry plots represent data of three independent experiments.

Next, we questioned whether bacteria that were not detectable anymore using our flow cytometry settings in Fig 1 were completely disintegrated, or whether their shape changed in such a way that they appeared differently in the FSC/SSC plots. To track the changes in FSC/SSC of serum-exposed bacteria, we labeled their LPS with a DBCO-Cy3 dye through click-chemistry with a metabolically incorporated KDO-azide (**Fig 2A**)[24]. In addition, we lowered the SSC threshold in our flow cytometry settings, to detect smaller particles in the samples. Cy3-labeled bacteria were exposed to 5% serum or Δlysozyme serum with or without 5 μg/ml purified lysozyme, after which the number of Cy3-positive particles was quantified within a fixed volume. In contrast to the particle loss we observed in Fig 1, the total number of Cy3-positive events remained similar in all conditions (**Fig 2B**). This suggests that the total number of bacterial particles detected by the flow cytometer is not altered by incubation with serum. When we further analyzed the FSC/SSC of these Cy3-positive particles, we again observed that bacteria exposed to serum have an altered size and shape, as evidenced by a shift in FSC/SSC (**Fig 2C**), which was less pronounced in the absence of lysozyme. The fact that the shift in scattering pattern of bacteria treated with Δlysozyme serum is more pronounced than in Fig 1, may be explained by the labeling procedure these bacteria have undergone, which may render them more susceptible for membrane damage by the MAC and other serum components (**Figs 1A** and **2C**). The number of Cy3-positive particles inside the FSC/SSC gate drastically decreased when 5 μg/ml lysozyme was added to the Δlysozyme serum (**Fig 2C** and **2D**). Altogether, these data show that the combination of the MAC and lysozyme in serum triggers alterations in the shape and size of *E*. *coli*, which we observe by a FSC/SSC shift compared to non-treated bacteria. Although these bacteria are not completely disintegrated, the cell wall is damaged in such a way that it drastically changes the bacterial morphology.

### The combination of the MAC and lysozyme alters the cell shape of *E*. *coli* from rod-shaped to spherical

**Fig 3:**
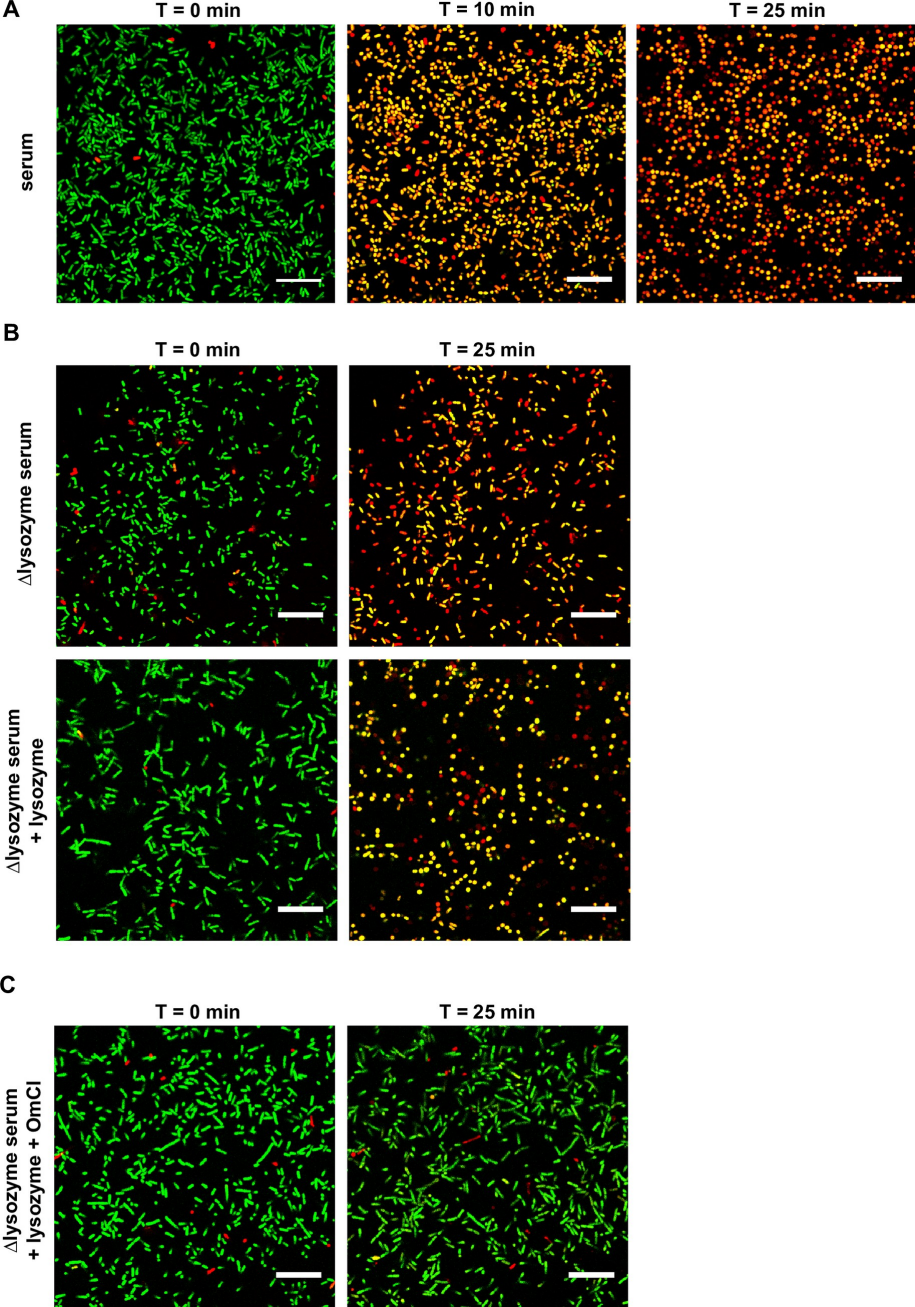
The combination of the MAC and lysozyme alters the cell shape of *E*. *coli* from rod-shaped to spherical. Confocal microscopy images of _Peri_mCherry/_cyto_GFP *E*. *coli* bacteria that were immobilized onto poly-L-lysin coated coverslips and treated with **A**) 5% serum or **B**) 5% Δlysozyme serum with (bottom) or without (top) 0.5 μg/ml lysozyme. All incubations were done in the presence of To-pro-3 as a readout for inner membrane damage. Images were taken after **A**) 0, 10 and 25 minutes or **B**) 0 and 25 minutes at 37°C. In **C**), bacteria were treated with 5% Δlysozyme serum with 0.5 μg/ml lysozyme in the presence of 20 μg/ml OmCI to block MAC formation. **B, C**) The lysozyme concentration that was used exceeds the concentration in 5% serum by a factor 10. **A-C**) Scale bars: 20 μm. Images represent data of three independent experiments.

To further validate our observations by flow cytometry, we aimed to visualize the effect of serum on the morphology of *E*. *coli* in more detail by confocal microscopy. We treated _peri_mCherry/_cyto_GFP *E*. *coli* with serum and analyzed their morphology in time. We added an impermeable DNA dye (ToPro-3) to monitor inner membrane destabilization. Control bacteria that were exposed to buffer remained ToPro-3 negative and almost all bacteria were classified as rod-shaped (**S2A and S2B Fig**). The inner membrane of serum-treated bacteria was efficiently damaged after approximately 10 minutes. After 25 minutes of serum exposure, the cell shape of almost all imaged bacteria had changed from rod-shaped to spherical, suggesting that also the peptidoglycan layer was affected (**Figs 3A** and **S2B**). We subsequently addressed the role of lysozyme in these experiments by exposing bacteria to 5% Δlysozyme serum with and without 0.5 μg/ml purified lysozyme. Although we observed inner membrane damage in Δlysozyme serum within 25 minutes, there was no significant decrease in the number of rod-shaped bacteria compared to the buffer control (**Figs 3B** and **S2B**). The small decrease in the number of cells that was classified as rod-shaped is in line with the flow cytometry observations where Δlysozyme serum causes slight alterations in the scattering pattern (**Fig 1C**). Noteworthy, although a higher percentage of bacteria that were treated with Δlysozyme serum was classified as non-rod-shaped, many of these bacteria have an intermediate phenotype. This could potentially be caused by other serum proteins and peptides that can enter through MAC pores or by leakage of periplasmic proteins. In contrast, when bacteria were exposed to Δlysozyme serum that was supplemented with purified lysozyme, a significant number of the imaged bacteria lost their rod-shape within 25 minutes (**Figs 3B** and **S2B**). Lysozyme-dependent alterations in cell shape were also dependent on the presence of MAC pores, since there was no decrease in the percentage of rod-shaped bacteria in the presence of the C5 inhibitor OmCI (**Figs 3C** and **S2B**). To better visualize the differences in cell shape of these bacteria, 3D reconstructions were generated of the conditions described in Fig 3B (exposed for 45 minutes at room temperature, **S2C Fig**). Altogether, these confocal images confirm that the MAC in serum allows lysozyme to trigger alterations in the shape of *E*. *coli* from rod-shaped to spherical.

### A completely assembled MAC pore sensitizes *E*. *coli* to the antimicrobial actions of lysozyme and hGIIA

**Fig 4:**
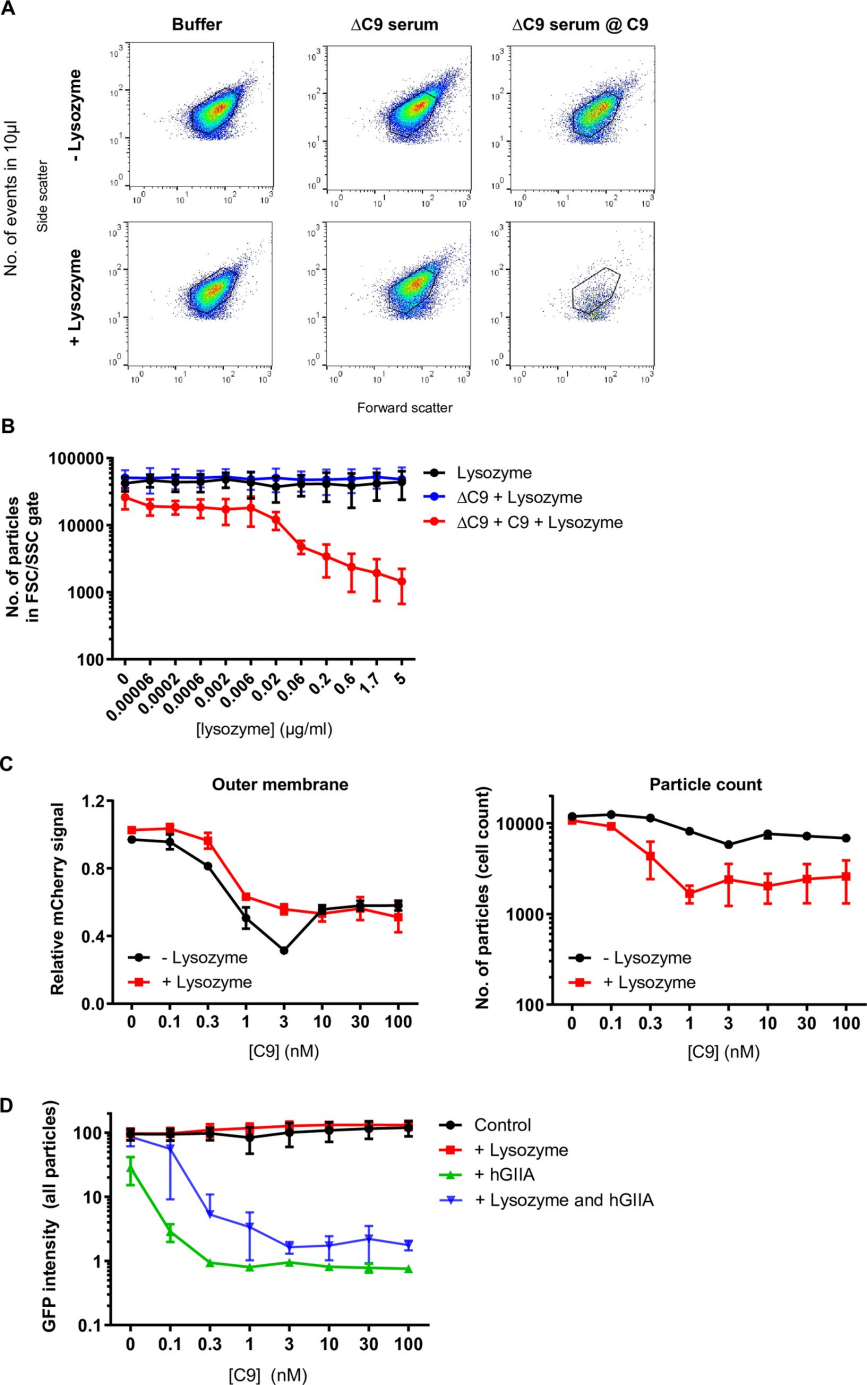
A completely assembled MAC pore sensitizes *E*. *coli* for cell wall degradation by lysozyme and hGIIA. **A**) Flow cytometry plots (FSC/SSC) and **B**) particle count of _Peri_mCherry/_cyto_GFP *E*. *coli* that was pre-treated with buffer or 10% ΔC9 serum and, after washing (indicated with an @), exposed to buffer, 100 nM C9 and/or a concentration range of lysozyme for 60 minutes. In **A**, the flow cytometry plots of 0 and 5 μg/ml lysozyme are depicted. **B**) A gate was set on untreated bacteria as depicted in **A**, after which the number of particles was counted within those gates. **C**) Outer membrane damage (left: mCherry signal (relative to the mean of the mCherry signals of the 0 nM C9 controls)) and particle count (right) of _Peri_mCherry/_cyto_GFP *E*. *coli* that were treated with 10% ΔC9 serum, and after washing, exposed to a concentration range of C9 in the presence or absence of 5 μg/ml lysozyme for 60 minutes. Particle count represents the number of particles in the FSC/SSC gate of untreated bacteria. **D**) GFP intensity of _Peri_mCherry/_cyto_GFP *E*. *coli* that was pre-treated with 10% ΔC9 serum and, after washing, exposed to a concentration range of C9 in the absence (control) or presence of 5 μg/ml lysozyme and/or 1 μg/ml hGIIA for 30 minutes. The GFP intensity (Geomean) of the total number of events is presented. **A**) Flow cytometry plots represent data of three independent experiments. **B-D**) Data represent mean ±SD of 3 independent experiments.

Next, we investigated whether a complete MAC pore is required for further destruction of the cell envelope by other immune factors. Bacteria were pre-incubated with C9-depleted serum (ΔC9 serum), leading to C5b-8 complex formation on the bacterial surface. C5b-8-labeled bacteria were washed, after which purified C9 was added in the presence or absence of a concentration range of lysozyme. A minor increase in Sytox was observed when bacteria were treated with ΔC9 serum alone, indicating that C5b-8 complexes already slightly damage the bacterial inner membrane (**S3A Fig**). Although the bacterial inner membrane was more efficiently damaged in the presence of C9 (**S3A Fig)**, there were hardly any alterations in FSC/SSC detected (**Fig 4A** and **4B**). Lysozyme by itself did not trigger inner membrane damage or alterations in the FSC/SSC on bacteria with C5b-8 complexes (**Figs 4A, 4B and S3A**). In contrast, the number of particles inside the FSC/SSC gate decreased when lysozyme was added in combination with C9 (**Fig 4A** and **4B**). To validate whether outer membrane damage by the MAC is responsible for the lysozyme-dependent particle loss, we next titrated C9 onto bacteria with C5b-8 complexes in the presence or absence of 5 μg/ml lysozyme, and monitored mCherry leakage and particle loss simultaneously. Indeed, lysozyme-induced particle loss coincided with the leakage of mCherry from the periplasm (**Fig 4C**).

Next, we investigated whether the MAC also sensitizes *E*. *coli* to immune factors with a different mechanism of action. Therefore, we included an antimicrobial protein that hydrolyzes the cytoplasmic membrane of bacteria, Type IIA secreted phospholipase A2 (hGIIA)[21]. Similar to lysozyme, hGIIA cannot cross the outer membrane of Gram-negative bacteria and is therefore considered to act specifically against Gram-positive bacteria [25,26]. Since hGIIA acts on the cytoplasmic membrane, we now also included leakage of cytoplasmic GFP as a readout for inner membrane damage. To prevent full particle loss in conditions with the MAC and lysozyme, we shortened the final incubation step with C9 and lysozyme to 30 minutes, leading to a shift in FSC/SSC but no complete loss of particles. This allowed us to simultaneously study the effect of these two antibacterial proteins on alterations in bacterial morphology and levels of cytoplasmic GFP (**S3B Fig**). Despite the alterations in FSC/SSC of bacteria that were treated with C9 and lysozyme, these bacteria remained GFP positive (**Fig 4D**). This suggests that lysozyme did not affect the integrity of the inner membrane. In line with previous observations, the MAC alone did also not affect the level of cytoplasmic GFP (**Fig 4D**)[10]. We then measured whether the MAC could also sensitize *E*. *coli* to 1 μg/ml recombinant hGIIA (250x excess compared to non-inflamed conditions or comparable to the concentration in inflamed serum [27,28]). Although some particles were lost from the FSC/SSC gate when bacteria with C5b-8 complexes were exposed to hGIIA, the effect was much more substantial when hGIIA was combined with C9 (**S3B and S3C Fig**). Bacteria that were still detected after treatment with a combination of hGIIA and C9 appeared within the FSC/SSC of untreated bacteria (**S3B Fig**), but were all GFP negative (**Fig 4D**), indicating that their inner membrane was severely damaged by hGIIA. We also observed a small drop in GFP signal when hGIIA was added to bacteria with C5b-8 complexes in the absence of C9, suggesting that some hGIIA can enter the periplasmic space through C5b-8 complexes in the outer membrane (**Fig 4D**; 0 nM C9 condition). A decrease in GFP signal was also observed when physiologically relevant concentrations of hGIIA (6 ng/ml) were used in combination with C9 (**S3D Fig**). As expected, almost all events were lost when bacteria were treated with a combination of C9, lysozyme and hGIIA (**S3B and S3C Fig**). Altogether, we conclude that the MAC sensitizes *E*. *coli* to antimicrobial proteins with different effector functions, lysozyme acting on the peptidoglycan layer and hGIIA on the bacterial inner membrane.

### The MAC sensitizes *E*. *coli* to killing by neutrophils

**Fig 5:**
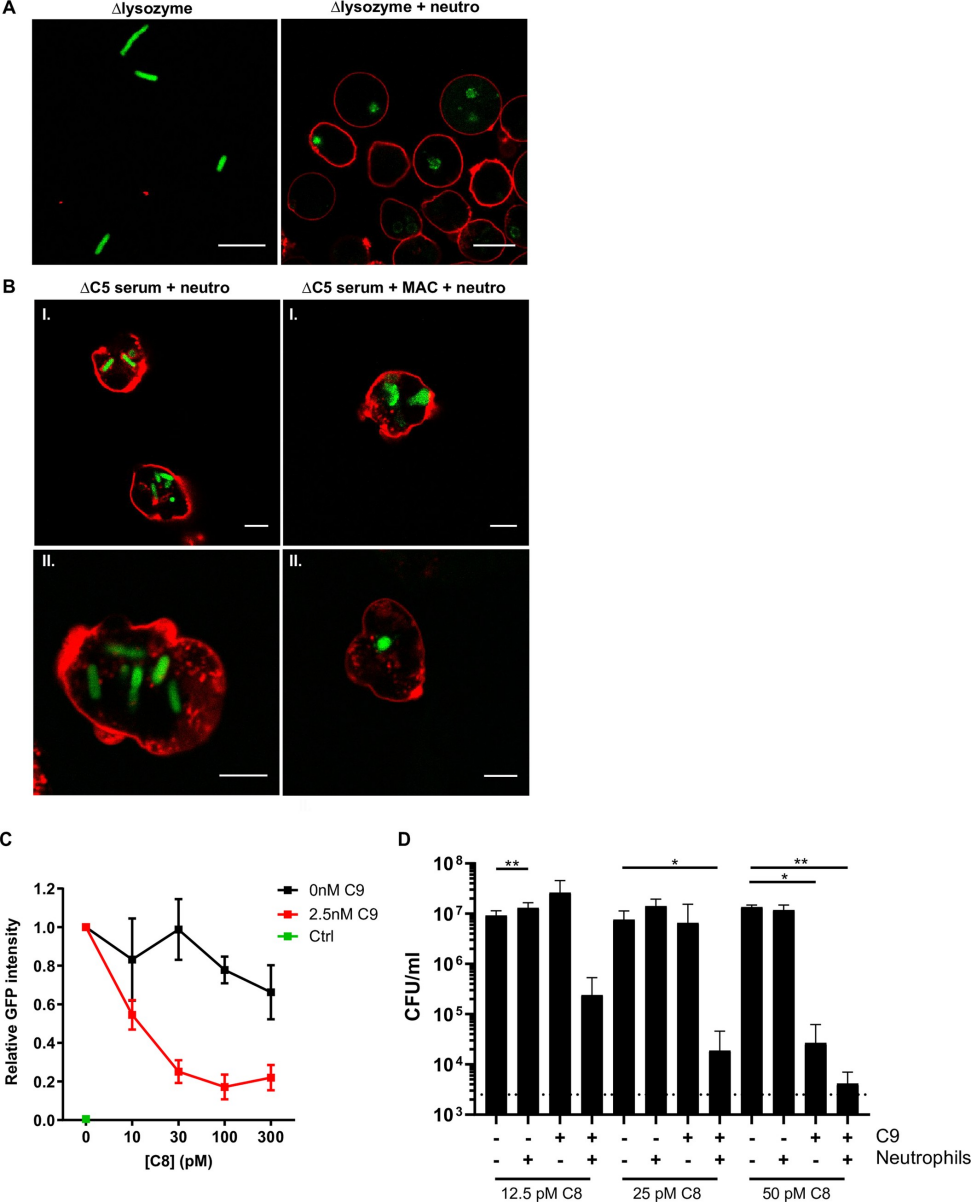
The MAC sensitizes *E*. *coli* to killing by neutrophils. **A**) Confocal microscopy images of _Peri_mCherry/_cyto_GFP *E*. *coli* (green) treated with 5% Δlysozyme serum for 60 minutes at 37°C. After washing, bacteria were incubated with buffer (left) or neutrophils (red, right) for 20 minutes at 37°C, fixed in 1.5% paraformaldehyde and imaged. **B**) Confocal images of _Peri_mCherry/_cyto_GFP *E*. *coli* (green) that were pre-labeled with 10% ΔC5 serum (for deposition of C5 convertases), washed and exposed to buffer or C5-C9 for 30 minutes at 37°C. 10 nM C5 and C6, 20 nM C7 and C8 and 100 nM C9 was used. After washing, bacteria were exposed to neutrophils for 20 minutes at 37°C, fixed in 1.5% paraformaldehyde and imaged. **A, B**) Neutrophils membranes (red) were stained with Alexa-647 labeled Wheat Germ Agglutinin. **C**) Relative GFP intensity of neutrophils after phagocytosis of _Peri_mCherry/_cyto_GFP *E*. *coli*. Bacteria were pre-labeled with 10% ΔC8 serum and, after washing, exposed to a concentration range of C8 in the presence or absence of 2.5 nM C9 for 30 minutes at 37°C. After washing, bacteria were incubated with neutrophils for 20 minutes at 37°C, after which the GFP signal of the neutrophils was analyzed by flow cytometry. GFP intensity was normalized against the GFP intensity of bacteria that were treated with ΔC8 serum only. Neutrophils without bacteria (green dot) served as basal value for the GFP signal. **D**) Bacterial viability (CFU/ml) of *E*. *coli* that was pre-treated with MAC components similar to **C**, and exposed to buffer or neutrophils. After 20 minutes, neutrophils were lysed for 15 minutes in MQ, after which bacterial survival was determined (CFU/ml). **C**, **D**) Bacteria/neutrophil ratio used: 10/1. **A, B**) Images represent two (**A**) or four (**B**) independent experiments in which the 488-laser settings were adjusted to the GFP intensity. **C, D**) Data represent mean ±SD of 3 independent experiments. **D**) Statistical analysis was done using a ratio paired t-test in which each condition was compared to the buffer control (no C9 and no neutrophils) within the same C8 concentration. Significance was displayed only when significant as *P ≤ 0.05 or **P≤ 0.01.

For intracellular killing by neutrophils, Gram-negative bacteria are taken up into phagolysosomes that contain a large number of highly-concentrated antimicrobial proteins and peptides [16,29]. Several of these proteins, including lysozyme and hGIIA, are considered inactive against Gram-negative bacteria and require other factors in the phagolysosome (such as lactoferrin, defensins and bactericidal permeability increasing protein (BPI)), to first damage the bacterial outer membrane [12,30–33]. Given that the MAC efficiently sensitizes *E*. *coli* to antimicrobial proteins in serum, we hypothesized that it may also sensitize bacteria to antimicrobial factors inside neutrophils. To test this, we treated _Peri_mCherry/_cyto_GFP *E*. *coli* with Δlysozyme serum to allow MAC formation while maintaining cell shape (**Fig 5A**), and exposed them to human neutrophils to allow phagocytosis. Intracellular bacteria were efficiently degraded, as evidenced by the diffused GFP signal and the absence of clear rod shape bacteria (**Fig 5A**). Quantification of the number of rod-shaped versus non-rod-shaped bacteria inside the imaged neutrophils revealed that 100% of the imaged bacteria was non-rod-shaped (**S4A Fig**). In serum, bacteria are efficiently labeled with C3b molecules, which enhances phagocytosis and thereby facilitates bacterial killing by neutrophils [34,35]. However, the contribution of MAC pores to killing by neutrophils is less well understood. To directly study the effect of MAC pores on sensitizing bacteria to neutrophils, we incubated _Peri_mCherry/_cyto_GFP *E*. *coli* with ΔC5 serum to label the bacterial surface with C5 convertase as previously described [18]. These bacteria were then exposed to buffer, or to purified C5-C9 to allow pore formation. When convertase-labeled bacteria were internalized by neutrophils, 82% of the imaged bacteria remained rod-shaped within the measured time-frame (**Figs 5B** and **S4B**). Instead, when convertase-labeled bacteria were first exposed to purified MAC components (C5-C9) and then internalized by neutrophils, only 23% of the imaged bacteria remained rod-shaped (**Figs 5B** and **S4B**). Importantly, a higher laser intensity was needed to detect the GFP signal in the samples with MAC components and a lower number of round-shaped bacteria was imaged per neutrophil (**S4B Fig**). Besides the fact that GFP is more diffuse in these conditions, it might also be degraded inside the phagolysosome. To test whether a full MAC pore is required to sensitize bacteria to neutrophils, we included a condition in which C9 was omitted, to allow MAC formation up to C8. When these bacteria were phagocytosed, approximately 60% of the imaged bacteria remained rod-shaped. The other 40% of the imaged bacteria became spherical or had an intermediate phenotype (**S4B and S4C Fig**). This suggests that incomplete MAC pores (C5b-8) can also partly sensitize *E*. *coli* to (small) intracellular factors. One of these factors could be hGIIA, that has some activity on bacteria with C5b-8 pores (**Fig 4D**).

We next performed flow cytometry experiments to study the role of the MAC inside neutrophils in a more high-throughput manner. We carefully titrated the number of MAC pores on _Peri_mCherry/_cyto_GFP bacteria by exposing them to ΔC8 serum and, after washing, a concentration range of C8 in the presence or absence of C9. Low concentrations of C8 and C9 were added to trigger outer membrane damage of *E*. *coli* by the MAC, without inducing killing (**Fig 5C and 5D**). These bacteria were subsequently exposed to neutrophils. The GFP signal within the neutrophil population drastically decreased when pre-labeled bacteria were incubated with both C8 and C9. In contrast, it remained relatively stable when only C8 was added (**Fig 5C**). The decrease in GFP signal when bacteria were pre-treated with full MAC pores also explains why a higher 488-laser intensity was required in the confocal experiments to detect GFP inside neutrophils in these conditions. We next repeated the experiment with bacteria of which the LPS was labeled with Cy3 to verify that the efficiency of phagocytosis was unaffected by the pre-treatment with different combinations of MAC components. Although there is a slight drop in Cy3 signal of the neutrophil population when bacteria were pre-treated with C8 and C9, this effect is minimal compared to the loss of GFP signal, suggesting that the drop of GFP is not caused by impaired phagocytosis (**Figs 5C and S4D**). This also suggests that, due to the loss of GFP signal, we were unable to image all bacteria by confocal microscopy, since we counted a lower number of bacteria inside the neutrophils when these were pre-treated with full MAC pores (**S4B Fig**). Finally, we used the same experimental setup to assess whether the MAC also increases bacterial killing (CFU/ml) by neutrophils. When only C8 was added to bacteria pre-treated with ΔC8 serum, no killing was observed in the presence or absence of neutrophils (**Fig 5D**). At the highest concentration of C8 (50 pM), *E*. *coli* was efficiently killed in the presence of C9, and therefore only a slight additive effect of neutrophils could be measured (**Fig 5D**). However, at the C8 concentrations where addition of C9 or neutrophils alone was not sufficient to kill these bacteria (12.5 and 25 pM), the combination of both triggered efficient killing of these bacteria (**Fig 5D**). Altogether, these results show that the MAC enhances cell wall degradation and killing of *E*. *coli* inside neutrophils.

## Discussion

Infections with Gram-negative bacteria form an increasing problem for human health, which can partly be attributed to the presence of an outer membrane that is selectively permeable to antibiotics and endogenous antimicrobials. Therefore, combination therapy of antibiotics and outer membrane permeabilizing agents has become more attractive over the last decades [5–9]. Additionally, our own immune system can damage the bacterial outer membrane in such a way that it sensitizes bacteria to naturally ineffective antibiotics. Specifically, MAC pores efficiently disrupt the bacterial outer membrane, allowing antibiotics to enter the periplasmic space and fulfill their antimicrobial functions [10]. Our recently developed fluorescent reporter system enabled us to unravel in detail how the complement system kills Gram-negative bacteria. These tools are essential to discriminate between different types of membrane damage and to study how these influence bacterial viability and cell wall integrity. By labeling different cell compartments and the bacterial cell membrane (LPS), we are now able to study membrane damage and shape changes by flow cytometry and confocal microscopy. Using these tools, we previously demonstrated that the MAC forms pores in the bacterial outer membrane that trigger destabilization of the bacterial inner membrane, which is essential for bacterial killing. However, we noticed that the complement system alone does not affect the cell morphology of Gram-negative bacteria [18], suggesting that other factors are required for further cell wall degradation. Here we show that the MAC plays a crucial role in sensitizing Gram-negative bacteria to other human immune factors, such as antimicrobial proteins in- and outside phagocytes.

One of these factors is lysozyme, an antimicrobial protein that degrades peptidoglycan. Using flow cytometry, we observed that the MAC and lysozyme in serum are both required to trigger alterations in the morphology of *E*. *coli*. These findings were verified by confocal microscopy, in which we observed that the MAC and lysozyme together affect the cell wall in such a way that *E*. *coli* bacteria change from rods to spheres. These findings correlate with previous electron microscopy studies, showing that the MAC can kill bacteria, but that a combination of the MAC and lysozyme damages the bacterial cell wall more severely [36–39]. The peptidoglycan layer of Gram-negative bacteria is essential for the integrity of the bacterial cell wall and to withstand turgor pressure from the cytoplasmic space [40]. Degradation of this layer by lysozyme could therefore explain why these bacteria lose their rod-shaped morphology. To the best of our knowledge, lysozyme is the only factor in serum that affects the bacterial peptidoglycan layer, however we cannot exclude that other serum components may also contribute to the drastic morphological changes in serum in a lysozyme dependent manner. Although lysozyme is generally known as an antimicrobial protein against Gram-positive bacteria, several Gram-negative species (among which *E*. *coli* and *Neisseria gonorrhoeae*) have evolved resistance mechanisms against these enzymes [41–43]. The fact that lysozyme plays a crucial role in cell wall degradation of Gram-negative bacteria in the presence of the complement system may explain why some of these bacteria have developed evasion strategies. Since the *E*. *coli* strain that we used is efficiently killed by the MAC alone, the additional effect of lysozyme on killing of this strain is probably limited and difficult to quantify. Therefore, it remains to be elucidated how this effect translates to less MAC sensitive bacteria in an *in vivo* setting. However, we propose that the observed synergy between complement and other immune factors could apply to a broader range of Gram-negative bacteria, especially to those on which the MAC can damage the outer membrane, but where these pores are not able to directly kill the bacteria [10].

The MAC also sensitizes *E*. *coli* to hGIIA, which hydrolyzes phospholipids in bacterial membranes [21]. A combination of MAC pores and hGIIA triggered efficient leakage of cytoplasmic proteins and a loss of particles detected by flow cytometry. The fact that MAC pores in the outer membrane allow both lysozyme and hGIIA to severely damage the bacterial cell envelope suggests that this principle applies to a broad range of antimicrobial proteins that are considered inactive against Gram-negative bacteria. Lysozyme and hGIIA are both secreted by epithelial cells and immune cells that are recruited towards the site of infection [13,21]. As determined in this study, the concentration of lysozyme in 100% non-inflamed serum is approximately 1–2 μg/ml. However, the lysozyme concentrations in serum and other bodily fluids increases under inflammatory conditions [44]. To the best of our knowledge, there are no studies available that directly compare the lysozyme concentration in serum of healthy donors and serum of patients that have an infection with a Gram-negative bacterium. The normal serum concentration of hGIIA is approximately 4 ng/ml [45,46] and can increase up to 1 μg/ml in inflamed serum [27,28]. Inside immune cells, the concentrations of these proteins may be even higher than in inflamed serum. Although the concentrations of these proteins have been measured in bodily fluids in “inflamed conditions”, it remains challenging to determine what concentrations would be relevant to mimic the conditions at the site of infection or inside immune cells. These will most probably vary between individuals, depend on the location of the infection and on the immune status of the patient. Although we show that concentrations of lysozyme and hGIIA that are physiologically relevant in non-inflamed serum can synergize with MAC pores, this study should still be considered as a proof of principle, since it is difficult to directly translate these findings to infection conditions in the human body.

Bacteria can also be internalized by immune cells. Phagocytosed bacteria are subsequently exposed to high concentrations of intracellular lysozyme, hGIIA and other antimicrobial peptides and proteins in the phagolysosome. Among these proteins are lactoferrin, defensins and bactericidal permeability increasing protein (BPI), which can all enhance outer membrane permeability [12,30–33]. The complement system enhances phagocytosis of Gram-negative bacteria by depositing C3b on the surface, which interacts with complement receptors on the neutrophil surface [34,35]. However, within the time-frame of our experiments, internalized C3b-labeled bacteria were not degraded or killed by neutrophils, suggesting that this process is relatively inefficient. In contrast, internalized bacteria were efficiently degraded and killed when these were pre-treated with full MAC pores (C5b-9) that allow intracellular proteins to cross the outer membrane [18]. Since the tested bacterial strain is sensitive to killing by MAC pores, the experiment was set up in such a way that *E*. *coli* was not killed by the MAC alone to proof the principle of synergy between complement and neutrophils using our fluorescent bacteria. The synergy between the MAC and neutrophils may be more crucial for bacteria on which the MAC can damage the outer membrane, but not the inner membrane. These results suggest that the complement system does not only enhance phagocytosis via the deposition of C3b molecules, but that the MAC also plays a crucial role in degradation of Gram-negative bacteria by neutrophils. Vice versa, intracellular or secreted antimicrobial proteins that enhance outer membrane permeability of bacteria (such as BPI) may also enhance the efficiency with which MAC pores insert into the bacterial outer membrane and kill bacteria [9]. It is likely that several factors in the phagolysosome, among which lysozyme and hGIIA [19] are involved in the killing of MAC-opsonized bacteria. However, since killing of *E*. *coli* by neutrophils is only enhanced in the presence of all MAC components, it likely depends on proteins that need to cross the outer membrane.

We hypothesize that MAC-dependent degradation of the bacterial cell envelope by lysozyme, hGIIA and other antimicrobial proteins may facilitate and accelerate clearance of these particles from the body, for example by neutrophils. Since the shape of internalized particles influences immune cell activation [47] and more efficient degradation of bacteria inside immune cells may improve antigen presentation, this may also play a role in stimulation of a proper adaptive immune response by antigen-presenting cells. Faster clearance of Gram-negative bacteria from the body could prevent ongoing immune activation on the surface of these bacteria that are already killed. However, further research is needed to show the exact relevance of each step of cell envelope degradation on immune-mediated clearance of bacteria.

The fact that MAC-dependent outer membrane damage sensitizes Gram-negative bacteria to cell envelope degradation by lysozyme and hGIIA suggests that also other outer membrane permeabilizing agents can synergize with human immune factors. This study serves as a proof of principle showing that the MAC sensitizes *E*. *coli* to the antimicrobial actions of lysozyme, hGIIA and neutrophils. However, we propose that this mechanism may also be relevant for a broad range of other antimicrobial proteins, peptides and antibiotics that normally fail to pass the outer membrane of Gram-negative bacteria [10,48]. The synergy between MAC pores and antimicrobial proteins might even be more crucial to kill bacterial strains on which MAC pores damage the outer membrane but fail to damage the inner membrane and kill these bacteria.

## Materials and methods

### Ethics statement

Human blood was isolated after informed consent was obtained from all subjects in accordance with the Declaration of Helsinki. Approval was obtained from the medical ethics committee of the UMC Utrecht, The Netherlands.

### Serum and reagents

Pooled normal human serum (NHS) was obtained from healthy volunteers as previously described [49]. Human neutrophils were isolated on the day of the experiment from freshly drawn heparinized blood of healthy volunteers using density gradient centrifugation [50]. Complement factor C8 and sera depleted of C5, C8 or C9 were obtained from Complement Technology. C5, C6, C7 with a C-terminal and C9 with an N-terminal 6x His-tag were cloned into a UPE expression vector for recombinant protein expression in HEK293E cells (U-protein Express) and purified from the supernatant using immobilized metal affinity chromatography. OmCI was expressed and purified as previously described [23]. Lysozyme-depleted serum was prepared by affinity depletion using an LprI column and checked for complement activity, as described in [18]. The concentration of lysozyme in serum was determined by a lysozyme ELISA (Abcam). Human neutrophil lysozyme was obtained from RayBiotech. Heat inactivated lysozyme was obtained by leaving it for 1h at 95°C. To obtain _Peri_mCherry/_cyto_GFP *E*. *coli*, MG1655 was transformed with a pPerimCh plasmid, containing a constitutively expressed periplasmic mCherry and a L-arabinose inducible cytosolic GFP [18]. GFP expression was induced using 0.1% L-arabinose. Recombinant hGIIA was kindly provided by Gérard Lambeau (Université Côte d'Azur)[51]. KDO-azide was synthesized according to a reported procedure [52].

### LPS labeling via click-chemistry

A single colony of *E*. *coli* MG1655 was grown to stationary phase in Lysogeny Broth (LB) medium. Bacteria were subcultured by diluting 1/100 in LB medium in the presence of 2 mM KDO-azide and incubated overnight (o/n), shaking at 37°C. The next day, bacteria were washed three times in RPMI 1640 medium (Thermofisher) containing 0.05% human serum albumin (RPMI-HSA) and incubated with 50 μM DBCO-Cy3 for 2.5 hours, shaking at 4°C. Bacteria were washed three times in RPMI-HSA and resuspended in RPMI-HSA at OD_600_ ~0.1.

### Flow cytometry and bacterial viability assay

_Peri_mCherry/_Cyto_GFP *E*. *coli* were grown o/n in LB medium in the presence of 100 μg/ml ampicillin. The next day, subcultures were grown to mid-log phase (OD_600_~0.5) in the presence of 0.1% L-arabinose, washed and resuspended in RPMI-HSA. Bacteria of OD_600_~0.025 were mixed with buffer, NHS or Δlysozyme serum with or without lysozyme (concentrations indicated in the figure legend). For assays using purified complement components, bacteria were pre-treated with 10% ΔC5, ΔC8 or ΔC9 serum for 30 minutes at 37°C, washed three times and further incubated with buffer or the remaining MAC components for 30 or 60 minutes, as indicated in the figure legend. Also the concentrations of purified MAC components are specified in the figure legends. When indicated, a concentration range, or 5 μg/ml lysozyme or 1 μg/ml hGIIA was added to these incubations. One μM Sytox blue (ThermoFisher) was used to measure inner membrane damage. The number of particles, or the GFP, Sytox blue or green, mCherry or Cy3 intensity was measured by a MACSQuant flow cytometer (Miltenyi Biotech). For this, either a fixed volume (10 μl) or a fixed number of particles (10.000) was measured, with or without a SSC threshold, as indicated in the figure legends. To determine bacterial viability, samples were serially diluted into PBS and plated onto LB agar plates. Colonies were counted after overnight incubation.

### Neutrophil phagocytosis and killing assay

_Peri_mCherry/_Cyto_GFP *E*. *coli* MG1655 or Cy3-labeled WT *E*. *coli* MG1655 were treated as described in the figure legends, and incubated with neutrophils at a bacteria/neutrophil ratio of 10:1 to allow phagocytosis for 20 minutes shaking at 37°C. For neutrophil phagocytosis assays, samples were diluted twenty times in RPMI + 0.05% HSA after which the GFP or Cy3 signal of a fixed number of neutrophils (10^5^) was analyzed by flow cytometry (MACSQuant, Miltenyi Biotech). To determine bacterial viability, samples were diluted 1:20 in MilliQ and incubated for 15 minutes to allow neutrophil lysis. Afterwards, samples were serially diluted into PBS and plated onto LB agar plates. Colonies were counted after overnight incubation.

### Confocal microscopy

For the time-course experiments, _Peri_mCherry/_Cyto_GFP *E*. *coli* were grown in the presence of L-arabinose as described above, washed three times in PBS and concentrated to OD_600_~1.2 in PBS. Bacteria were immobilized on a poly-L-lysine (0.01%, Sigma-Aldrich) covered 8 well μ-slide chamber (Ibidi) for 45 minutes. Chambers were rinsed three times with PBS after which RPMI-HSA containing 1 μM To-pro-3 (Thermofisher) was added. A T = 0 image was taken, after which 5% normal serum or Δlysozyme serum with or without 0.5 μg/ml purified lysozyme was added. The Δlysozyme serum + lysozyme was imaged with and without 20 μg/ml OmCI. GFP and To-pro-3 intensity was measured after the indicated time-points at 37°C or when indicated at RT. To image GFP bacteria inside neutrophils, the samples were prepared as described above and fixed in 1.5% paraformaldehyde. Neutrophil membranes were stained for 15 minutes with 2 μg/ml Alexa Fluor 647-conjugated Wheat Germ Agglutinin (ThermoFisher). Samples were concentrated and dried onto 1% agar pads. All images were obtained using a Leica SP5 confocal microscope with a HCX PL APO CS 63×/1.40–0.60 OIL objective (Leica Microsystems, the Netherlands). GFP was measured using the 488 laser, Alexa-647 and To-pro-3 were imaged using the 647 laser. Both lasers were used in combination with the appropriate emission filter settings. The number of rod versus round shaped bacteria was quantified by hand in a blinded way.

### Data analysis and statistics

Flow cytometry data was analyzed using FlowJo (version 10). Graphpad 6.0 was used for graph design and statistical analysis. Statistical analysis was done as indicated in the figure legends. Three experimental replicates were performed to allow statistical testing. Confocal images were processed in Fiji.

## Supporting information

S1 Fig Lysozyme in serum is not essential for bacterial killing, but enhances membrane disintegration in the presence of the MAC. **A**) Lysozyme ELISA standard curve and **B**) Lysozyme ELISA to determine the concentration of lysozyme in serum and Δlysozyme serum. The arrows in **B** indicate the measurements that were interpolated into the standard curve (**A**) to determine the lysozyme concentration in the two sera (see S1 Table). **C**) *E*. *coli* cell count in 10 μl after exposure to a concentration range of serum or Δlysozyme serum with or without 5 μg/ml lysozyme in the presence or absence of 20 μg/ml OmCI for 60 min 37°C. Flow cytometry settings were similar to Fig 1A and 1B. **D**) Bacterial viability (CFU/ml) of *E*. *coli* exposed to buffer, 1% serum with or without 20 μg/ml OmCI or Δlysozyme serum. **A, B**) Graphs represent data of three independent experiments. **C, D**) Data represent mean ±SD of 3 independent experiments.
(TIF)

S2 Fig The MAC and lysozyme alter the cell shape of *E. coli* from rod-shaped to spherical. **A)** Confocal microscopy image of _Peri_mCherry/_cyto_GFP *E*. *coli* bacteria that were immobilized onto poly-L-lysin coated coverslips and treated with RPMI for 25 minutes with a similar experimental setup as in Fig 3. **B**) Quantification of the percentage of rod-shaped bacteria within each of the conditions depicted in Fig 3. The total number of quantified bacteria is mentioned for each condition. **C**) 3D reconstructions of confocal microscopy images of _Peri_mCherry/_cyto_GFP *E*. *coli* bacteria that were immobilized onto poly-L-lysin coated coverslips. A T = 0 image was taken, after which bacteria were exposed to 5% Δlysozyme serum with or without 5 μg/ml lysozyme. All incubations were in the presence of To-pro-3 as a readout for inner membrane damage. Images were taken after 45 minutes at room temperature. Scale bars: **A**) 20 μm, **C**) 10 μm. **B**) Data represents mean ±SD of quantifications of 3 independent confocal experiments per condition. Statistical analysis was done using an unpaired t-test in which each condition was compared to the buffer control. Significance was displayed only when significant as *P ≤ 0.05 or **P ≤ 0.01.
(TIF)

S3 Fig **A**) Inner membrane damage (Sytox blue intensity) of _Peri_mCherry/_cyto_GFP *E*. *coli* that were pre-treated with buffer or 10% ΔC9 serum and, after washing (indicated with an @), exposed to buffer, 100 nM C9 or 5 μg/ml lysozyme. **B**) Flow cytometry plots (FSC/SSC) of _Peri_mCherry/_cyto_GFP *E*. *coli* that was pre-treated with 10% ΔC9 serum and, after washing (indicated with an @), exposed to buffer or 100 nM C9 in the presence or absence of 5 μg/ml lysozyme and/or 1 μg/ml hGIIA for 30 minutes. **C**) Particle count of _Peri_mCherry/_cyto_GFP *E*. *coli* that was pre-treated with 10% ΔC9 serum and, after washing, exposed to a concentration range of C9 in the absence (control) or presence of 5 μg/ml lysozyme and/or 1 μg/ml hGIIA for 30 minutes. A gate was set on untreated bacteria, after which the number of particles was counted within those gates. **D**) GFP intensity (Geomean of all particles) of _Peri_mCherry/_cyto_GFP *E*. *coli* that was pre-treated with buffer or 10% ΔC9 serum and, after washing exposed to buffer (ctrl) or 6 ng/ml hGIIA in the presence or absence of 100 nM C9 for 30 minutes. **A**, **B**) Histograms and flow cytometry plots represent data of three independent experiments. **C, D**) Data represent mean ±SD of 3 independent experiments. **D**) Statistical analysis was done using a ratio paired t-test in which each condition was compared to the buffer control. Significance was displayed only when significant as *P ≤ 0.05.
(TIF)

S4 Fig The MAC does not influence phagocytosis of *E. coli* by neutrophils, but does influence intracellular degradation. **A, B)** Quantification of the percentage of rod shaped versus non-rod shaped bacteria within the conditions depicted in Figs 5A and 5B and **S4**C. The total number of counted bacteria within the total number of imaged neutrophils is mentioned for each condition. **C**) Confocal images of _Peri_mCherry/_cyto_GFP *E*. *coli* (green) that was pre-labeled with 10% ΔC5 serum (for deposition of C5 convertases), washed and exposed to C5-C8 for 30 minutes at 37°C. After washing, bacteria were exposed to neutrophils (conditions comparable to Fig 5B). Images represent data of two independent experiments. **D**) Cy3 intensity (relative to buffer control) of neutrophils after phagocytosis of DBCO-Cy3-labeled *E*. *coli*. Bacteria were exposed to 10% ΔC8 serum for 30 minutes at 37°C and washed. Bacteria were subsequently incubated with buffer, 0.03 nM C8, 2.5 nM C9 or a combination of both for 30 minutes at 37°C. After washing, bacteria were incubated with neutrophils for 20 minutes at 37°C. Cy3 intensity within the neutrophil population was analyzed by flow cytometry. Data represent mean ±SD of 3 independent experiments. Statistical analysis was done using a paired t-test in which each condition was compared to the buffer control. Significance was displayed only when significant as **P ≤ 0.01.
(TIF)

S1 Table Lysozyme concentration in serum and Δlysozyme serum. The lysozyme concentration in normal serum and Δlysozyme serum was determined by a lysozyme ELISA as depicted in S1 Fig. The arrows in S1B Fig. indicate the measurements that were interpolated into the standard curve of S1A Fig. (0.01%, 0.03% and 0.1% for normal serum and 1.1%, 3% and 10% for Δlysozyme serum). The lysozyme concentration in 100% serum was determined for these measurements, after which the average of the three measurements was calculated.
(TIF)
