# Supplementary figures and images for: Outer membrane permeabilization by the membrane attack complex sensitizes Gram-negative bacteria to antimicrobial proteins in serum and phagocytes
Source: PLoS Pathog. 2021 Jan 22;17(1):e1009227. doi: 10.1371/journal.ppat.1009227 (PMC7886145; doi:10.1371/journal.ppat.1009227)

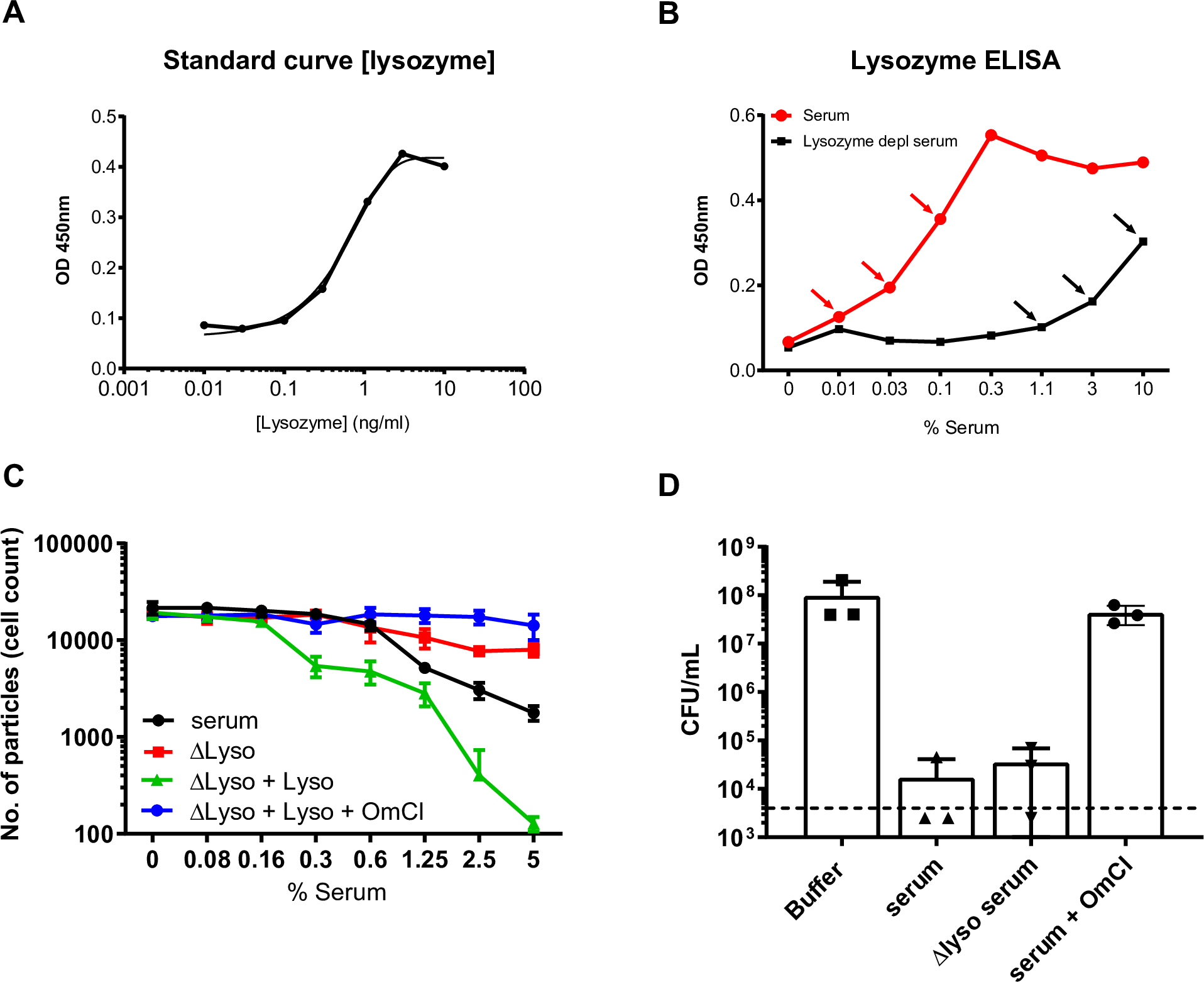

Supplement: S1 Fig — A) Lysozyme ELISA standard curve and B) Lysozyme ELISA to determine the concentration of lysozyme in serum and Δlysozyme serum. The arrows in B indicate the measurements that were interpolated into the standard curve (A) to determine the lysozyme concentration in the two sera (see S1 Table). C) E. coli cell count in 10 μl after exposure to a concentration range of serum or Δlysozyme serum with or without 5 μg/ml lysozyme in the presence or absence of 20 μg/ml OmCI for 60 min 37°C. Flow cytometry settings were similar to Fig 1A and 1B. D) Bacterial viability (CFU/ml) of E. coli exposed to buffer, 1% serum with or without 20 μg/ml OmCI or Δlysozyme serum. A, B) Graphs represent data of three independent experiments. C, D) Data represent mean ±SD of 3 independent experiments. (TIF) [file ppat.1009227.s001.tif]

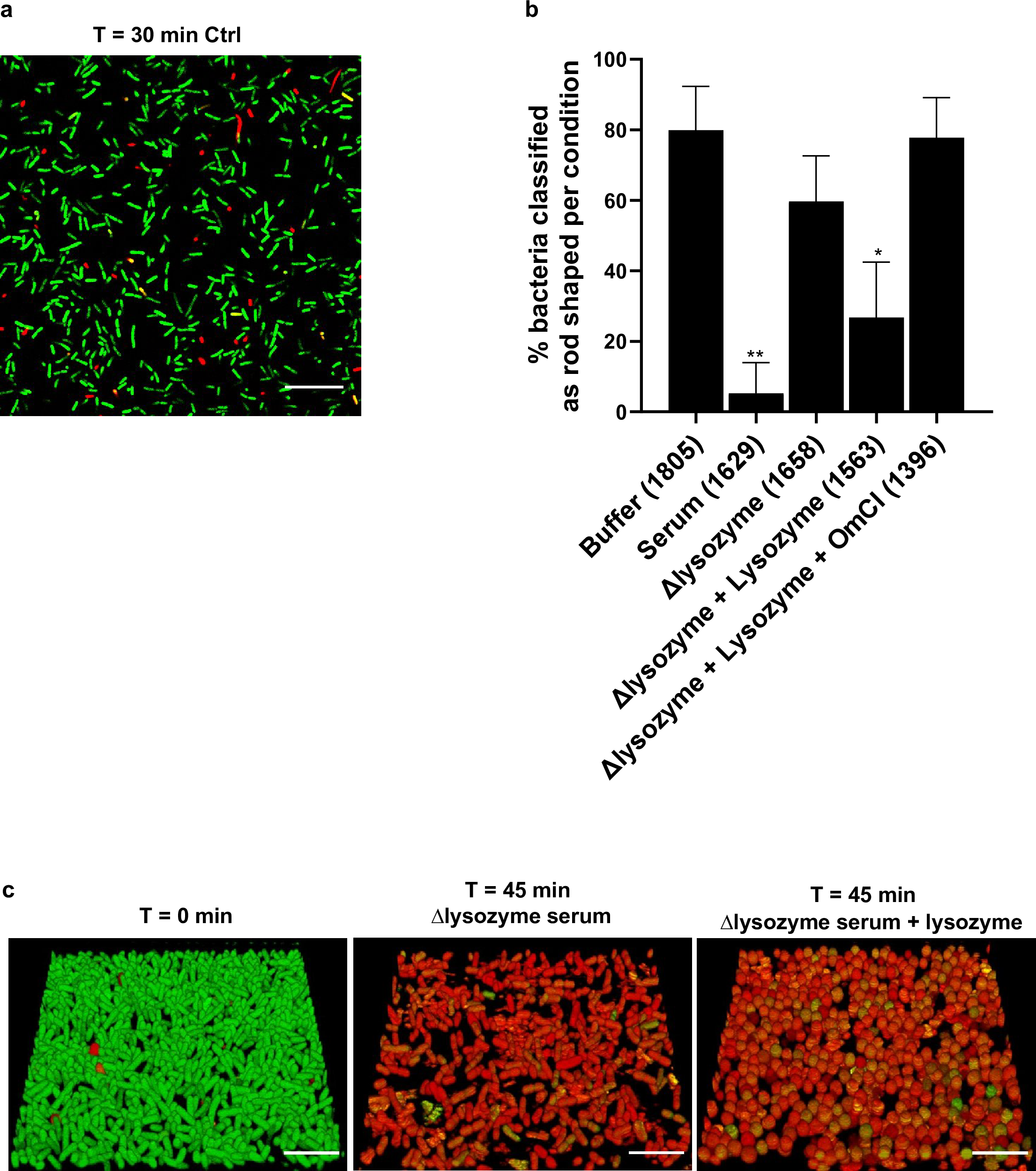

Supplement: S2 Fig — A) Confocal microscopy image of PerimCherry/cytoGFP E. coli bacteria that were immobilized onto poly-L-lysin coated coverslips and treated with RPMI for 25 minutes with a similar experimental setup as in Fig 3. B) Quantification of the percentage of rod-shaped bacteria within each of the conditions depicted in Fig 3. The total number of quantified bacteria is mentioned for each condition. C) 3D reconstructions of confocal microscopy images of PerimCherry/cytoGFP E. coli bacteria that were immobilized onto poly-L-lysin coated coverslips. A T = 0 image was taken, after which bacteria were exposed to 5% Δlysozyme serum with or without 5 μg/ml lysozyme. All incubations were in the presence of To-pro-3 as a readout for inner membrane damage. Images were taken after 45 minutes at room temperature. Scale bars: A) 20 μm, C) 10 μm. B) Data represents mean ±SD of quantifications of 3 independent confocal experiments per condition. Statistical analysis was done using an unpaired t-test in which each condition was compared to the buffer control. Significance was displayed only when significant as *P ≤ 0.05 or **P ≤ 0.01. (TIF) [file ppat.1009227.s002.tif]

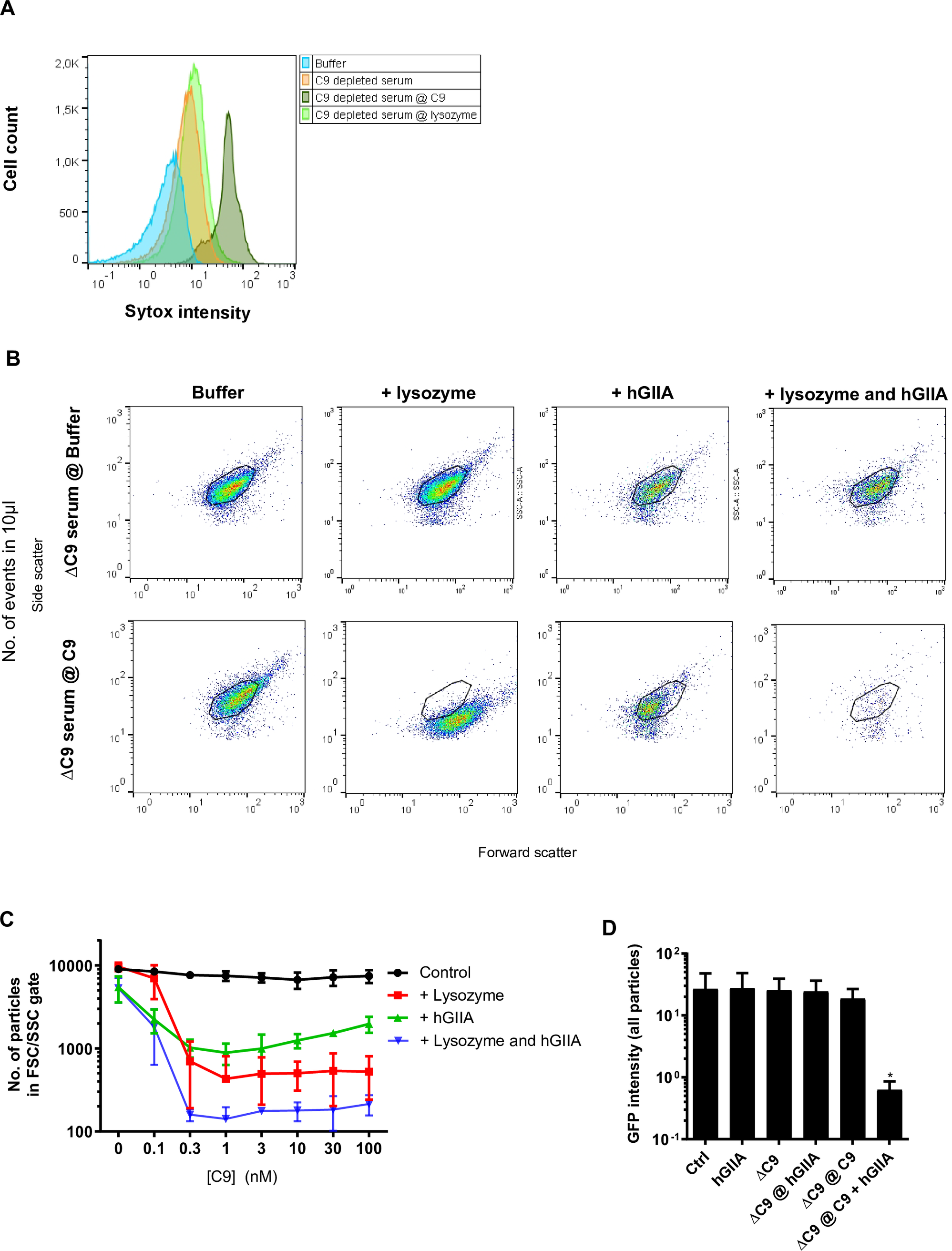

Supplement: S3 Fig — A) Inner membrane damage (Sytox blue intensity) of PerimCherry/cytoGFP E. coli that were pre-treated with buffer or 10% ΔC9 serum and, after washing (indicated with an @), exposed to buffer, 100 nM C9 or 5 μg/ml lysozyme. B) Flow cytometry plots (FSC/SSC) of PerimCherry/cytoGFP E. coli that was pre-treated with 10% ΔC9 serum and, after washing (indicated with an @), exposed to buffer or 100 nM C9 in the presence or absence of 5 μg/ml lysozyme and/or 1 μg/ml hGIIA for 30 minutes. C) Particle count of PerimCherry/cytoGFP E. coli that was pre-treated with 10% ΔC9 serum and, after washing, exposed to a concentration range of C9 in the absence (control) or presence of 5 μg/ml lysozyme and/or 1 μg/ml hGIIA for 30 minutes. A gate was set on untreated bacteria, after which the number of particles was counted within those gates. D) GFP intensity (Geomean of all particles) of PerimCherry/cytoGFP E. coli that was pre-treated with buffer or 10% ΔC9 serum and, after washing exposed to buffer (ctrl) or 6 ng/ml hGIIA in the presence or absence of 100 nM C9 for 30 minutes. A, B) Histograms and flow cytometry plots represent data of three independent experiments. C, D) Data represent mean ±SD of 3 independent experiments. D) Statistical analysis was done using a ratio paired t-test in which each condition was compared to the buffer control. Significance was displayed only when significant as *P ≤ 0.05. (TIF) [file ppat.1009227.s003.tif]

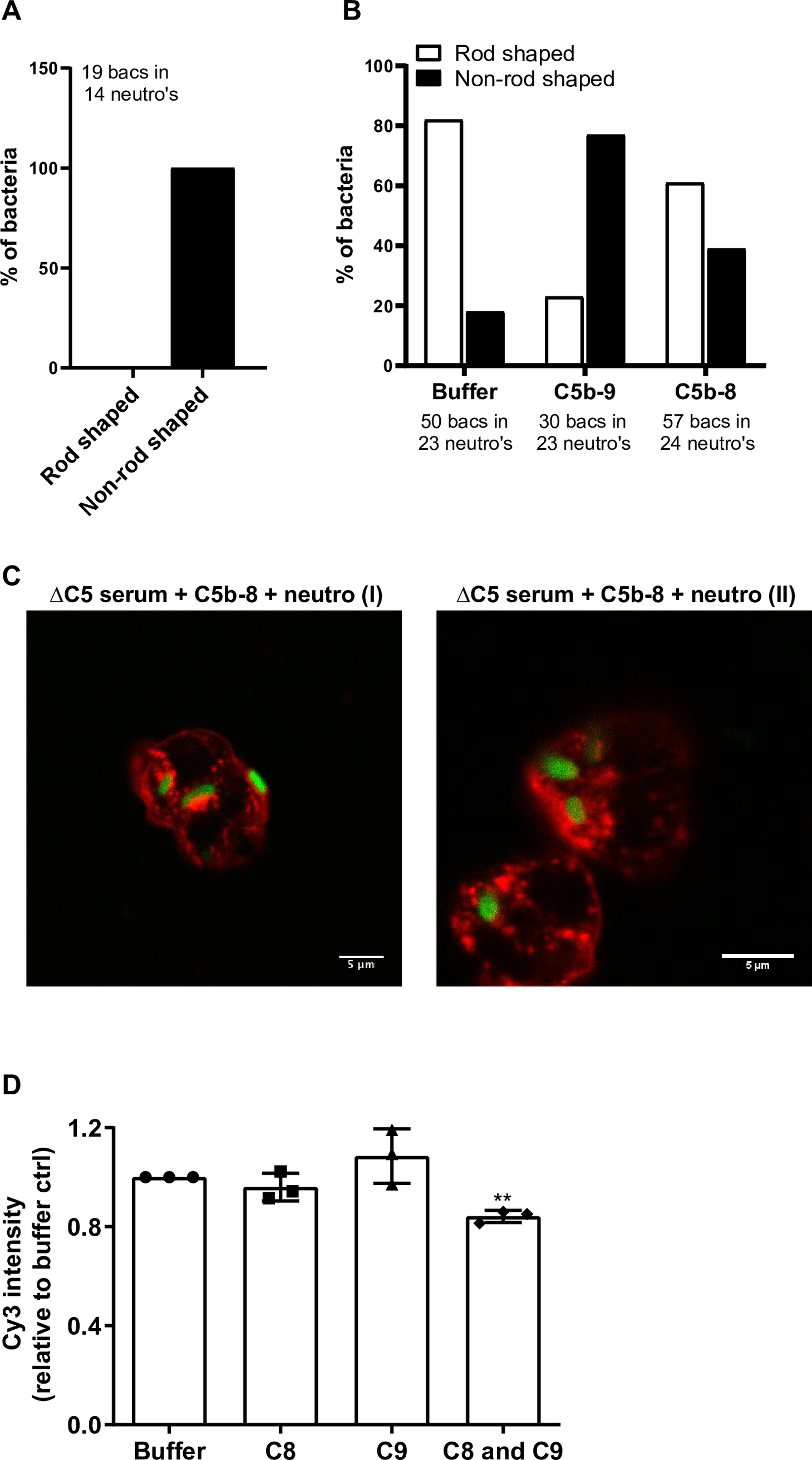

Supplement: S4 Fig — A, B) Quantification of the percentage of rod shaped versus non-rod shaped bacteria within the conditions depicted in Figs 5A and 5B and S4C. The total number of counted bacteria within the total number of imaged neutrophils is mentioned for each condition. C) Confocal images of PerimCherry/cytoGFP E. coli (green) that was pre-labeled with 10% ΔC5 serum (for deposition of C5 convertases), washed and exposed to C5-C8 for 30 minutes at 37°C. After washing, bacteria were exposed to neutrophils (conditions comparable to Fig 5B). Images represent data of two independent experiments. D) Cy3 intensity (relative to buffer control) of neutrophils after phagocytosis of DBCO-Cy3-labeled E. coli. Bacteria were exposed to 10% ΔC8 serum for 30 minutes at 37°C and washed. Bacteria were subsequently incubated with buffer, 0.03 nM C8, 2.5 nM C9 or a combination of both for 30 minutes at 37°C. After washing, bacteria were incubated with neutrophils for 20 minutes at 37°C. Cy3 intensity within the neutrophil population was analyzed by flow cytometry. Data represent mean ±SD of 3 independent experiments. Statistical analysis was done using a paired t-test in which each condition was compared to the buffer control. Significance was displayed only when significant as **P ≤ 0.01. (TIF) [file ppat.1009227.s004.tif]

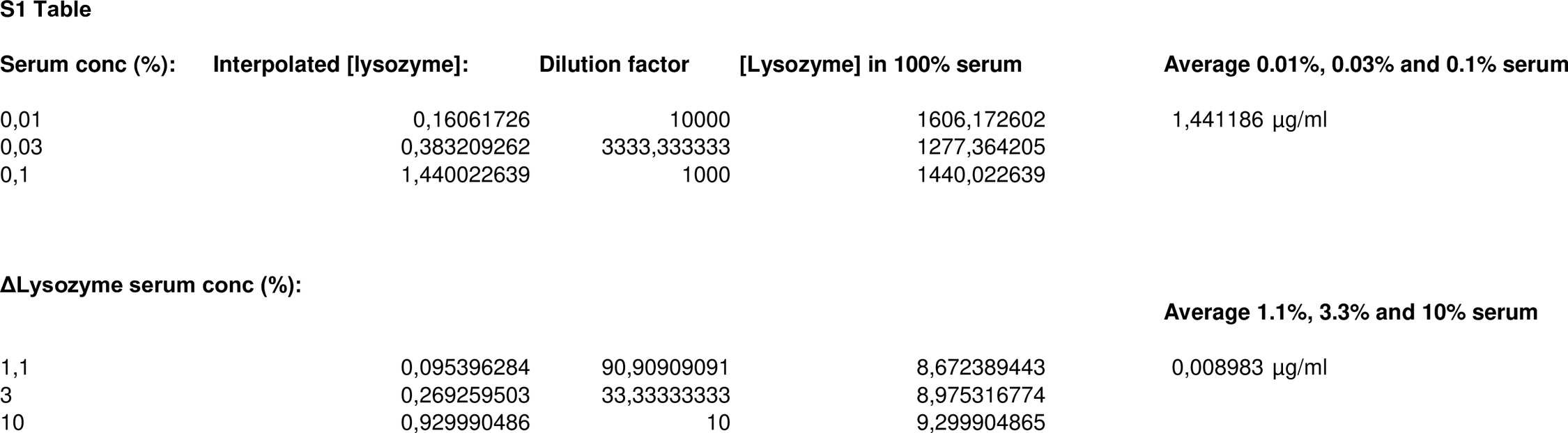

Supplement: S1 Table — The lysozyme concentration in normal serum and Δlysozyme serum was determined by a lysozyme ELISA as depicted in S1 Fig. The arrows in S1B Fig. indicate the measurements that were interpolated into the standard curve of S1A Fig. (0.01%, 0.03% and 0.1% for normal serum and 1.1%, 3% and 10% for Δlysozyme serum). The lysozyme concentration in 100% serum was determined for these measurements, after which the average of the three measurements was calculated. (TIF) [file ppat.1009227.s005.tif]
