# Supplementary material for: Outer membrane permeabilization by the membrane attack complex sensitizes Gram-negative bacteria to antimicrobial proteins in serum and phagocytes
Source: PLoS Pathog. 2021 Jan 22;17(1):e1009227. doi: 10.1371/journal.ppat.1009227 (PMC7886145; doi:10.1371/journal.ppat.1009227)
Supplement: Attachment — Submitted filename: 103327_1_rebuttal_1907757_qj9n6t.docx [file ppat.1009227.s006.docx]

**Response to reviewers**

We kindly thank the reviewers for carefully reading our paper and for providing feedback on how to improve the manuscript. Our revised manuscript contains 10 new subfigures, 8 subfigures with (small) revisions, a new supplementary table and textual adjustments in order to improve manuscript as suggested by the reviewers. Please find below our point-to-point reply to the reviewers’ concerns.

**Reviewer #1:**

**Reviewer 1 summarizes the study as follows and emphasizes its relevance:**

*The authors suggest that the function of Complement MAC complexes in the outer membrane of Gram negative bacteria extends beyond membrane destabilisation. Instead the pores could allow for access of large, proteinogenic factors of the immune response to the peptidoglycan layer and inner membrane, rendering the bacteria susceptible to a much broader array of antimicrobrial proteins as well as neutrophil mediated killing. In this sense the MAC would be much more similar to the perforin system which has been proposed to support the membrane passage of granzymes (cf. Spicer et al., Seminars in Cell & Developmental Biology 72 (2017) 117-123) than the current literature model suggests. Notably, perforin pores are made up from the same fold as the complement MAC pores. The concept of a protein conducting channel formed as a result of the complement cascade is highly intriguing, and would be of great interest to a broad scientific community. The results from the underlying study definitely support that MAC formation can - under certain conditions - enhance the effects of antimicrobial proteins and immune cells.*

**However, the reviewer also raised some issues*:***

*However, as the authors allude in the discussion, the protein concentrations used in the study do not necessarily correlate with the concentrations under physiological conditions (e.g. using a 100x amount of lysoyme in the repleted serum compared to NHS levels, use of complement components in the picomolar range to study effects of neutrophil killing). Therefore, the study should be viewed as a successful proof-of-principle which yet needs to be investigated more thoroughly in order to probe its physiological relevance.*

**Answer:** **We added several new figures to the paper in order to improve these points raised by the reviewer. For a detailed description, please see the point-to-point reply below.**

**Major comments:**

**Point 1**

*In their initial experiments, the authors observe a change in bacterial morphology upon exposure of bacteria to normal human serum (NHS) and demonstrate that MAC formation is required for morphological changes (Fig 1-3).* *They propose lysozyme as the serum component which is responsible for the changes. This conclusion is based on the data shown in Figs 2 and 3 where bacteria are incubated in buffer (=untreated), NHS, lysozyme-depleted serum or lysozyme-depleted serum which has been supplemented with purified lysozyme (repleted serum). The changes in morphology are analysed either by flow cytometry (changes in scattering, Fig 2) or confocal microscopy (changes in cell-shape, Fig 3).*

***Question****: If lysozyme were to be the sole component responsible for the morphological changes, one would expect the lysozyme-depleted sample to be equivalent to untreated bacteria. However, Fig 2c still reveals a shift in scattering pattern with lysozyme-depleted serum compared to untreated bacteria, although the effect is less drastic compared to incubation in NHS. Can the authors provide an explanation for this observation?*

**Answer:** **We agree that in figure 2 there are changes in scatters when bacteria are treated with lysozyme-depleted serum. This shift is much more pronounced than in figure 1, probably because these bacteria were pre-labeled with KDO-azide and DBCO-Cy3 which requires an overnight incubation with KDO-azide and an additional labeling step with DBCO-Cy3. These steps may render the bacteria more vulnerable to membrane damage. We think these changes are caused by other antimicrobial serum proteins (such as hGIIA) that can enter through MAC pores in the outer membrane. We now clarify this in the results section of the revised manuscript (Line 209-212).**

**Point 2**

*Question A. Similarly, a significant number of round cells seems to be present in the confocal images shown for lysozyme depleted serum in Fig 3b at T=40 min as well as the 3D visualisation in Sfig 2. This is in direct contradiction to the claim "although we observed efficient inner membrane damage in delta-lysozyme serum within 40 minutes, these bacteria remained rod-shaped within the measured time-frame."*

**Answer A: We have now quantified the percentage of rod-shaped bacteria versus non-rod-shaped bacteria in the new images of three independent experiments (SFig. 2b). We also included a buffer control for statistical testing. Although not significant, the number of bacteria that is classified as rod-shaped after treatment with ∆lysozyme serum indeed slightly decreases when compared to the buffer control. This is in line with our flow cytometry observations, where we observed a small drop in particle count ∆lysozyme serum condition. Noteworthy, the bacteria that were classified as non-rod-shaped upon treatment with ∆lysozyme serum mostly have an intermediate phenotype, potentially caused by the leakage of periplasmic proteins or the influx of serum proteins and peptides through MAC pores. We clarified this more clearly in the manuscript (line 172-173, 209-212 and 237-240).**

*Question B. Making direct comparisons between the different incubation conditions is difficult because of the different time-frames used. However it appears that there are a similar proportion of round to rod shaped cells in the 40 minute delta-lysozyme sample as in the 60 minute NHS sample, which runs counter to the claim. In order to provide more support for the authors' conclusion it would be desirable to compare all conditions (NHS, lysozyme-depleted, lysozyme-repleted, lysozyme-repleted + MAC inhibitors) at identical time points and attempt for a quantification of round vs. rod-shaped cells present in the confocal images.*

**Answer B: We agree with the reviewer that for direct comparison, the same time-frames are required. Therefore, all conditions were now imaged at the same time-points (Revised Figure 3). In addition, these experiments were now performed at 37°C (instead of RT) to allow more direct comparison to the flow cytometry experiments in Figure 1.**

*Question C. In both experimental approaches, the effect on morphology is most drastic with repleted serum. However, it should be noted in the results section that the lysozyme concentration in these samples exceeds the concentration in the NHS samples by 100-fold (see l. 159).*

**Answer C: Since the high concentration of lysozyme was a clear concern of the reviewer, we now also performed the following experiments:**

1. **Flow cytometry: titrated lysozyme into lysozyme-depleted serum and also observed particle loss at physiological concentrations of 0.05 µg/ml (similar to what is physiologically present in 5% serum), (Revised Figure 1c and line 161-166). Furthermore, we titrated lysozyme and added physiological concentrations of hGIIA in the purified conditions (Revised Fig. 4b and SFig. 3d).**
2. **Confocal microscopy: We now added 0.5 µg/ml lysozyme to the depleted serum instead of 5 µg/ml to be able to more directly compare this to 5% nhs conditions (revised figure 3)**

**Point 3**

*Question: Together, these observations demonstrate that MAC formation allows lysozyme access to the peptidoglycan layer resulting in changes in morphology. However, it cannot be ruled out that at physiological concentrations of lysozyme, other factors contribute to this effect.*

**Answer: To the best of our knowledge, there are no other peptidoglycan-degrading enzymes in serum than lysozyme. Therefore, we think that the drastic changes in morphology from rod-shaped to round-shaped that was shown with confocal microscopy is mainly dependent on lysozyme, which is supported by the lysozyme depletion experiments in which we do see a slight increase in bacteria with an intermediate phenotype but not of the clear round-shaped bacteria. However, we agree with the reviewer that other serum factors may also act on *E. coli* when both MAC and lysozyme are present. We added this to the text (Line 399-402).**

**Point 4**

*Question: It is also interesting to note that reduction in bacterial viability is purely dependent on MAC formation (Sfig1) and not further altered in presence of absence of lysozyme. This yields the question what is the functional relevance of lysozyme-dependent altered morphology in vivo.*

**Answer: For the studied bacterium (*E. coli* MG1655) the effect of lysozyme depletion on viability is indeed limited, because this bacterium is highly sensitive to killing by the MAC alone. We would like to emphasize that we used this genetically engineered bacterium to mechanistically show how complement can synergize with other immune proteins. The effect on killing can be larger for other Gram-negative bacteria in which the MAC damages the outer membrane but fails to kill these bacteria** (Heesterbeek et al., 2019a) **(emphasized in line 407-413). Lastly, as speculated in the discussion (line 466-469), we hypothesize that a shape change from rod-shaped to spherical and more efficient degradation of bacteria in immune cells may contribute to more efficient immune cell activation, antigen presentation and stimulation of the adaptive immune system. This could also be relevant for bacteria that are already killed by the MAC.**

**Point 5**

*The authors then ask the question whether MAC formation sensitizes Gram negative bacteria to other antimicrobial proteins, besides lysozyme. They can demonstrate a MAC dependent susceptibility to hGIIA, an inner membrane destabilising protein which cannot act on Gram-negative bacteria on its own (Fig 5).Having demonstrated that MAC formation can sensitize Gram negative bacteria to different antimicrobial proteins in serum, the authors seek to investigate whether this effect extends to bacteria inside of neutrophils. The data presented in Fig 6a can demonstrate convincingly that bacteria which have been exposed to the full range of complement proteins in serum are efficiently degraded inside neutrophils. The authors then block the terminal steps of the complement cascade to distinguish between opsonisation and MAC-formation effects (Fig 6b).*

*Question: Could the authors comment on the differences in red fluorescence patterns between the neutrophils in Fig 6a and 6b? The neutrophils in 6a appear as smooth, roundish shapes whereas they seem to be much more irregular in shape, with gaps in fluorescent signal in 6b. Besides, there seems to be significant amounts of intracellular red fluorescence for the neutrophils imaged in 6b.*

**Answer 5: We agree with the reviewer that the staining pattern of the neutrophils varies. Since neutrophils are first fixed with 1.5% PFA and then incubated with AF647-conjugated-WGA, the cells are fully permeabilized and all membranes should be stained.** **Noteworthy, when going through all the images, there is no consistent pattern of when the staining is smooth and when it is more irregular. However, it seems like activated neutrophils show more irregular staining, probably because of a more activated phagocytosis process. We included some extra images to give a better overview of the generated data (Fig. 5b and SFig. 4c).**

**Point 6**

*Question A: Besides, the authors' choice of images for figure 6b raises the question why the panels shown here display only one (centre and right panel) or two (left panel) neutrophils? In my understanding this cannot be readily explained by experimental limitations since the magnification and experimental treatment of neutrophils is not too dissimilar from Figure 6a, where ~10 neutrophils are visible in one image.*

**Answer A: We understand the concern of the reviewer about the number of cells displayed. Therefore, we added two graphs to the manuscript in which we quantified the number of round versus rod shaped bacteria inside all the neutrophils we imaged (Revised SFig. 4a, b). Furthermore, we included some extra images to give a better overview of the generated data (Fig. 5b and SFig. 4c).**

*Question B: An increased number of depicted neutrophil cells would make a much stronger case for the authors' conclusions regarding the contribution of MAC pores to neutrophil-mediated killing. This is especially true since the bacteria encapsulated in the two neutrophil cells shown in the left panel of Fig 6b look quite different to each other. The two bacteria in the upper cell appear as distinct rod shapes, encapsulated in neutrophil membrane as judged by the layer of red fluorescence around them. In contrast, the bacteria in the lower cell do not display the red outline and their overall shape seems more variable. A higher number of neutrophils imaged might help to clarify this.*

**Answer B: As explained in point 5, we do not understand why some bacteria are surrounded by a stained neutrophil membrane and others are not. We added some more images and quantifications to the figures to strengthen our claims about the contribution of MAC pores in neutrophil killing.**

**Point 7**

*Question: When the authors test for the contribution of neutrophils bacterial viability they observe an additive effects of MAC formation and phagocytosis which the authors describe for low concentrations of C8 (Fig. 6d). According to the methods section, the bacteria used in these experiments are isolated from neutrophils post phagocytosis by lysing the neutrophils through incubation in MilliQ water. It can therefore not be ruled out that the reduced viability at low C8 can indeed be attributed to phagocytotic effects or is rather due to osmotic forces acting on the bacteria after neutrophil lysis.*

**Answer 7: Bacteria that are treated with MAC pores are also exposed to MQ, which has no effect on their viability. Therefore, it can be ruled out that MQ lyses bacteria of which the MAC has damaged the outer membrane.**

***- Are the key conclusions convincing?***

*Reviewer: Key conclusions are justified in a broad sense, but the in vivo relevance needs to be clarified*

**Answer: We now added new experiments showing that our findings are also relevant for physiological concentrations of lysozyme and hGIIA (point 2). Furthermore, we better explain that we provide proof of concept on a laboratory *E. coli* strain (which is highly sensitive for MAC). We think that synergy between the MAC and lysozyme/hGIIA is relevant for killing of other bacterial strains (point 4). In addition, we speculate that shape changes and improved degradation of bacterial particles inside immune cells may contribute to better stimulation of an adaptive immune response (point 4).**

***- Should the authors qualify some of their claims as preliminary or speculative, or remove them altogether?***

*Reviewer: As in the above point, clarification that these experiments are a proof of principle needs to be given more prominence in the manuscript. This concept is mentioned once in the discussion.*

**Answer: We now clarified this more clearly throughout the revised manuscript**

***- Would additional experiments be essential to support the claims of the paper? Request additional experiments only where necessary for the paper as it is, and do not ask authors to open new lines of experimentation.***

*Reviewer: It would be good to see equivalent time-points in the confocal experiments.*

**Answer: We performed these experiments and added the data to the revised manuscript (Revised figure 3).**

***- Are the data and the methods presented in such a way that they can be reproduced?***

*Reviewer: Yes*

***- Are the experiments adequately replicated and statistical analysis adequate?***

*Reviewer: More quantification of the cell shape in the confocal experiment would be beneficial. This could be compared with the results of the flow cytometry analysis.*

**Answer: We added quantification of the confocal data to the revised manuscript (SFig. 2b and SFig. 4a, b).**

**Minor comments:**

***- Are prior studies referenced appropriately?***

*Reviewer: l. 436, missing reference for purification of labelled complement factors, ref 436 only refers to purification of OmCI*

**Answer: We added additional information on how the complement proteins were expressed and purified and indicated that only OmCI was produced as described in the reference (Line 493-496).**

***- Are the text and figures clear and accurate?***

*1. Reviewer: l. 157-159, this sentence is in contradiction to l. 374-375. Please clarify whether the lysozyme concentration in repleted serum equals 5 µg/mL in 100% repleted serum or under experimental conditions of 5% serum.*

**1. Answer: The concentration of lysozyme added was 5 ug/ml to 5% serum. This is a ~100x higher concentration than can be found in 5% normal human serum (thus the concentration that was used in the experiment). We clarified this in the revised manuscript (Line 161).**

*2. Reviewer: l. 260, should this read "combination of hGIIA and lysozyme" rather than "combination of hGIIA and C5"?*

**2. Answer: Here we describe that bacteria that are pre-labeled with C5b-8 pores are slightly sensitive to hGIIA alone (0 nM C9-point in this graph shows a slight drop in the number of particles), but more sensitive to hGIIA when we titrate in C9. We clarified this in the revised manuscript (line 291-295).**

**Reviewer #2**

***(Significance):***

*The manuscript uses very elegant methods to demonstrate the role of lysozyme and the phospholipase hGIIA in degrading peptidoglycan and the inner membrane following insertion of MAC. These studies are novel and provide exciting advances in our knowledge of how complement may synergize with other serum proteins to impact Gram negative bacteria. The question asked is significant and the methods novel and robust - however the use of non-physiological conditions in many assays makes one question the relevance of the key findings.*

***(Evidence, reproducibility and clarity):***

*Heesterbeek et al have performed very elegant studies to demonstrate the role of lysozyme and the phospholipase hGIIA in degrading peptidoglycan and the inner membrane following insertion of MAC. These studies are novel and provide exciting advances in our knowledge of how complement may synergize with other serum proteins to impact Gram negative bacteria.*

***Concerns of reviewer 2:***

*The work, as presented, has two major drawbacks that dampen my enthusiasm. The assays were performed using reagents at non-physiological concentrations and as such it difficult to judge relevance. Further, the authors previously reported that MAC alone, in the absence of lysozyme or hGIIA, was able to kill E. coli and that killing was not associated with changes in cell morphology or release of cytoplasmic contents. While it seems clear that lysozyme and hGIIA (at least in the concentrations used in the manuscript) can disrupt cell morphology in the presence of MAC, it remains unclear what role they play in killing E. coli under physiological conditions*

**Answer: We added new figures to the paper in order to improve these points raised by the reviewer. For a detailed description, please see the point to point reply below.**

**Major comments**

**Point 1**

*Lysozyme and hGIIA have been used in concentrations much higher than physiological. Sera depleted of lysozyme was repleted with lysosome to a concentration that is 100X the normal physiological concentration and normal sera was supplemented with hGIIA at 5000X the normal or 100X the inflamed amount.*

*The authors attempt to justify this in the discussion, lines 375-385, citing likely elevated concentrations during the acute phase response and / or in certain biological niches (e.g. inside cells) and noting that the experiments should be taken as "proof of principle". While I agree that the concentrations of these molecules are likely elevated in response to infection and in certain niches, there is no justification for the excessive concentrations used and there is no way to distinguish "proof of principle" from laboratory artifact. Depleting of lysozyme provides strong evidence that is plays a role, unfortunately the complementation part of this experiment is flawed by the concentration of lysozyme used.*

*Question: Do physiologically relevant concentrations of these lysozyme and hGIIA have any effect? If not, how high does one need to go before seeing an effect? A dose response study is essential to determine the minimal amount of each compounded needed to have effect and to allow readers to determine relevance or if this seems plausible in an inflammatory respose.*

**Answer 1: We agree with the reviewer that testing physiologically relevant concentrations of the mentioned proteins would be a nice addition to the manuscript. Please see answer 2C of reviewer #1 for a detailed description of these experiments.**

**Point 2**

*The authors have previously established that MAC killing does not "trigger leakage of cytoplasmic proteins" and that "MAC pores do not alter the cell shape of bacteria". In this study, the authors very eloquently look at how complement and serum act in concert to degrade both peptidoglycan and the cytoplasmic membrane to effect cell morphology.*

*Question: It is difficult to gauge the significance of degradation of peptidoglycan and / or membrane disruption in killing relative to that of MAC alone. One can assume that cytoplasmic membrane disruption is associated with cell death but in most assays the bacteria used in the assay are already dead from exposure to MAC. If MAC alone kills bacteria, what is the significance of peptidoglycan and / or cytoplasmic membrane degradation?*

**Answer 2:** **We would like to refer to answer 4 of reviewer #1 to answer this question.**

**Point 3**

*Question: Most of the work presented is done using 5% serum, a rather low concentration. Where might these amounts be physiologically relevant?*

**Answer 3: Although the serum concentration in the blood (100%) is relevant in situations of bacterial sepsis where bacteria manage to enter the bloodstream, most infections occur at localized sites of the body (urinary tract, nose, lungs, skin). Here, bacteria will encounter lower concentrations of serum than in whole blood. During an infection, the inflammatory response will alter the local concentration of complement proteins due to increased secretion by epithelial cells, vascular permeability and resultant leakage of serum proteins from blood tissue. For example, the C1q concentration in urine (in infection conditions) was found to be comparable to the concentration of 4.4% serum and the C3 concentration rises up to concentrations comparable to 1% serum (Li et al., 2008). This suggests that the concentration of 5% used in this system is a relevant concentration. In addition, this is the serum concentration to which the tested *E. coli* strain is sensitive, without adding an extreme overload of serum.**

**Point 4**

*The data presented in Figure 6d, does indicate that sub-lethal MAC may increase the sensitivity of E. coli to killing by neutrophils. The assay is a bit contrived using low amounts of serum and limiting amounts of C8 and C9.*

*Question: What amount of C8 and C9 would be physiological for 10% serum? Where might bacteria encounter 10% sera with limiting MAC? It appears in a natural situation MAC containing E. coli would be dead prior to internalization and the subsequent MAC dependent changes in morphology would occur after E. coli has already been killed - as such is this physiologically relevant?*

**Answer 4: We agree with the reviewer that the experiment was setup in such a way that we were able to show that MAC pores enhance intracellular killing as a proof of principle and that it is unlikely that this situation would occur *in vivo* on this strain. However, this setup was required to properly show this principle on the *E. coli* MG1655 strain with the fluorescent reporter system. Importantly, we previously described that the condition where bacteria have outer membrane damage but no inner membrane damage does happen on bacteria that are more resistant to the MAC** (Heesterbeek et al., 2019b)**. This makes them more sensitive to antibiotics and most likely also to killing by neutrophils. We now emphasized more clearly that this experiment serves as a proof of principle (line 449-453).**

**Minor comments:**

*1. Reviewer: The abstract should refer specifically to hGIIA rather than referring to phospholipases or antimicrobial peptides in general. The latter gives the impression that multiple compounds were tested when the study is specific to lysozyme and hGIIA only.*

**1. Answer: We changed this to hGIIA in abstract**

*2. Reviewer: All figure text (key, titles and legends) must specify the concentrations of the reagents used and clearly indicate if it was in excess of known physiological concentrations - for example Figure 1 should read dlysozyme + 100X lysozyme. Similarly, the % sera should be indicated in each figure as it changes between 1, 5 and 10%. Including this information is important given the use of non-physiological reagents, such as 100X lysozyme or 5000X hGIIA and 1% serum.*

**2. answer: We have now described more clearly throughout the text and in the figure legends what the concentrations of the proteins that we used are, and emphasized more clearly that we used an excess in the text and in several figures legends. In addition, we now also titrated lysozyme and added physiological concentrations of hGIIA to further validate our findings. In our opinion, it is difficult to add an excess factor to each figure, since the reference value is not always clear and varies between “normal” and “inflamed” conditions.**

*3. Reviewer: Please modify the statement made in line 233, "since no mCherry (26 kDa protein) leaks out through C5b-8 pores, we assume that no serum proteins can enter the periplasmic space at this stage." Although I agree that C5b-8 is unlikely to form a pore that allows serum proteins to enter the periplasm, failure of mCherry to leak out is not firm evidence of this. There are most certainly proteins smaller and / or more compact that mCherry in serum (e.g. antimicrobial peptides).*

**3. Answer: We removed this statement from the manuscript since we agree with the reviewer that other smaller and/or more compact proteins may be able to cross the outer membrane through C5b-8 pores. For example, we show in the manuscript that hGIIA has some activity on bacteria with C5b-8 pores.**

*4. Reviewer: Why are both Eculizumab and OmCI needed to block C5 in Figure 3 whereas OmCI alone was used in Figure 1? When both are used, is each added at 20 µg/ml? For clarity, please specify OmCI or OmCI + Eculizumab rather than using "MAC inhibitors".*

**4. Answer: Both OmCI and Eculizumab efficiently block C5 cleavage, however there are studies that show that a combination of both inhibitors more completely block C5 cleavage, which is why we added both inhibitors in the first place. However, since only OmCI is sufficient to block the effect in the FACS experiments, we repeated the confocal experiment with just OmCI (Figure 3C of revised manuscript).**

*5. Reviewer: Please check reference 18 in line 375, reference 18 does not appear to determine the normal physiological concentration of lysozyme to be 1 µg/ml - please provide a reference for this information.*

**5. Answer: We thank the reviewer for making this point, since we agree that although reference 18 shows a lysozyme ELISA, data is missing to conclude what the concentration is in our serum pool. Therefore, we added the full data-set to determine the lysozyme concentration in serum and ∆lysozyme serum to the supplementary figures of this manuscript (SFig. 1a and b)**

*6. Reviewer: Line 141 what % of "full sera"?*

**6. Answer: We changed this in the text, referring to the 5% serum condition in Figure 1a (please see line 142 of revised manuscript)**

*7. Reviewer: Line 157, added back at 100X excess - this must be stated throughout the manuscript*

**7. Answer: We more clearly emphasized that this is an 100x excess in line 161 of revised manuscript.**

*8. Reviewer: Line 217-218 - statements indicating that the dlysozyme sera reconstituted with lysozyme is "more efficient" that normal sera must clarify that the lysozyme is 100X that found in normal sera.*

**8. Answer: We lowered the concentration of lysozyme in the confocal experiment (Revised Fig. 3), and emphasized that this is still a 10x excess the figure legend.**

*9. Reviewer: Section beginning with line 249 - specify fold excess hGIIA used - here and all other locations*

**9. Answer: We added this information to the text (line 284-285)**

*10. Reviewer: Line 255, typo - the word bacteria is repeated*

**10. Answer: Corrected**

*11. Reviewer: Figure 4b, yellow histogram - is part of this figure missing?*

**11. Answer: Due to the revisions, we included histograms of a different experiment to show the same principle (SFig. 3a). We excluded the condition in which C9 and lysozyme were added to ∆C9 serum, since there were hardly any events detected and we do not mention this condition in the text.**

*12. Reviewer: Figure 5a - please show dC9 + hGIIA with no C9 and dC9 + lysozyme with no C9. As noted also add fold excess of added lysozyme and hGIIA*

**12. Answer: We added the dotplots to the revised figure (now SFig. 3b) and clearly added the fold-increase in the text.**

*13. Reviewer: Figure 5b and c, "both" is ambiguous, use 100X lysozyme + 5000X hGIIA or equivalent*

**13. Answer: We changed “both” to lysozyme + hGIIA and clearly indicated the excess of protein used in the text.**

*14. Reviewer: Line 277, clarify what "other factors" in the phagolysosome*

**14. Answer: We clarified that we here mean intracellular factors such as lactoferrin, defensins and BPI that increase the outer membrane permeability of Gram-negative bacteria (line 305-306).**

*15. Reviewer: Figure 5 - Was 10% dC9 sera is used? Please add this information to the figure & legend.*

**15. Answer: This is correct and we incorporated this into the figure legend of Figure 4 and SFigure 3.**

**Reviewer #3**

***Significance:***

*The work presented here expands our view on the role of complement in killing of Gram-negative bacteria. The concept of MAC being involved not only in direct killing but in sensitizing bacteria to other antimicrobial agents is novel. This study will be of great interest to the broad fields of microbiology and immunology. It will be of particular relevance in my fields of expertise which are complement biology and microbial complement evasion. While the technical aspects of the paper appear to be well performed, I do not have extensive background in flow cytometry-based approaches.*

***(Evidence, reproducibility and clarity (Required)):***

*In the current study, Heesterbeek and colleagues describe a role for the complement membrane attack complex (MAC) in sensitizing Gram-negative bacteria to the action of secreted antimicrobial factors. This study builds on the group's previously developed E. coli-based fluorescent reporter system to delineate inner and outer membrane damage caused by MAC. Using this system along with a chemically modified LPS readout, the investigators employ flow cytometry and microscopy-based experiments to convincingly demonstrate that lysozyme and hGIIA work cooperatively with MAC to degrade the cell wall. Using a combination of depleted and repleted sera, the authors elegantly demonstrate that MAC-induced membrane damage is required for changes in the size and shape of E. coli cells in the presence of lysozyme or hGIIA. The authors hypothesize that MAC formation on Gram-negative bacteria is relatively inefficient in direct killing but serves to sensitize the bacteria to the action of antimicrobial agents like lysozyme and hGIIA. Finally, the authors extend their observations into neutrophils where they show that the combined activity of MAC and lysozyme enhances the killing of phagocytosed bacteria.*

**Reviewer #3 raises 1 major point:**

**Point 1.**

*Question: The manuscript is beautifully written, the data presentation and figure presentation are logical, and the study design is well conceived. I have only one suggestion involving the use of an additional control that would further strengthen the study. The involvement of the MAC is well characterized and controlled by using various complement- depleted sera and complement inhibitors. It was logical to select lysozyme and hGIIA as models for antimicrobial activity that might be enhanced by MAC-induced membrane damage and the data are clear. However, a negative control on this side of the experiment, similar to those used on the complement side would be useful here. Perhaps a serum protein not expected to be involved in antimicrobial activity, or a more interesting control where the lysozyme depleted serum is titrated with a catalytically inactivated lysozyme mutant. One would expect the effect of lysozyme to be abolished with such a mutant if the activity seen here is related to its canonical muramidase activity.*

**Answer: We now added a heat-inactivated variant of lysozyme (incubated for 1h at 95 degrees) to the incubations, and confirmed that this has no activity in our assays (please see revised Figure 1c).**

**Minor comments:**

Text changes

1. Line 159: "depended" should be "dependent"

2. Line 255: "pre-labeled bacteria" the word "bacteria" should be removed.

3. Line 282: "efficient" should be "efficiently".

**We corrected these textual aspects**

**References**

Heesterbeek, D. A. C., Martin, N. I., Velthuizen, A., Duijst, M., Ruyken, M., Wubbolts, R., Rooijakkers, S. H. M., & Bardoel, B. W. (2019a). Complement-dependent outer membrane perturbation sensitizes Gram-negative bacteria to Gram-positive specific antibiotics. *Scientific Reports*, *9*(1), 1–10. https://doi.org/10.1038/s41598-019-38577-9

Heesterbeek, D. A. C., Martin, N. I., Velthuizen, A., Duijst, M., Ruyken, M., Wubbolts, R., Rooijakkers, S. H. M., & Bardoel, B. W. (2019b). Complement-dependent outer membrane perturbation sensitizes Gram-negative bacteria to Gram-positive specific antibiotics. *Scientific Reports*, *9*(1), 3074. https://doi.org/10.1038/s41598-019-38577-9

Li, K., Sacks, S. H., & Sheerin, N. S. (2008). The classical complement pathway plays a critical role in the opsonisation of uropathogenic Escherichia coli. *Molecular Immunology*, *45*(4), 954–962. https://doi.org/10.1016/j.molimm.2007.07.037
